# Host species and geographic location shape microbial diversity and functional potential in the conifer needle microbiome

**DOI:** 10.1186/s40168-025-02271-y

**Published:** 2025-10-30

**Authors:** Robert M. Bowers, Shayna Bennett, Robert Riley, Juan C. Villada, Iolanda Ramalho Da Silva, Tanja Woyke, A. Carolin Frank

**Affiliations:** 1 U.S. Department of Energy, Joint Genome Institute, Berkeley, CA 94720 USA; 2 Life and Environmental Sciences, University of California, Merced, CA 95343 USA; 3 Sierra Nevada Research Institute, University of California, Merced, CA 95343 USA

**Keywords:** Microbial ecology, Phyllosphere, Conifer, Metagenome, Mobile genetic elements

## Abstract

**Background:**

The aerial surface of plants, known as the phyllosphere, hosts a complex and dynamic microbiome that plays essential roles in plant health and environmental processes. While research has focused on root-associated microbiomes, the phyllosphere remains comparatively understudied, especially in forest ecosystems. Despite the global ecological dominance and importance of conifers, no previous study has applied shotgun metagenomics to their phyllosphere microbiomes.

**Results:**

This study uses metagenomic sequencing to explore the microbial phyllosphere communities of subalpine Western conifer needle surfaces from 67 trees at six sites spanning the Rocky Mountains, including 31 limber pine, 18 Douglas fir, and 18 Engelmann spruce. Sites span ~ 1,075 km and nearly 10° latitude, from Glacier National Park to Rocky Mountain Biological Laboratory, capturing broad environmental variation. Metagenomes were generated for each of the 67 samples, for which we produced individual assemblies, along with three large coassemblies specific to each conifer host. From these datasets, we reconstructed 447 metagenome-assembled genomes (MAGs), 417 of which are non-redundant at the species level. Beyond increasing the total number of extracted MAGs from 153 to 294, the three coassemblies yielded three large MAGs, representing partial sequences of host genomes. Phylogenomics of all microbial MAGs revealed communities predominantly composed of bacteria (*n* = 327) and fungi (*n* = 117). We show that both microbial community composition and metabolic potential differ significantly across host tree species and geographic sites, with site exerting a stronger influence than host.

**Conclusions:**

This dataset offers new insights into the microbial communities inhabiting the conifer needle surface, laying the foundation for future research on needle microbiomes across temporal and spatial scales. Variation in functional capabilities, such as volatile organic compound (VOC) degradation and polysaccharide metabolism, closely tracks shifts in taxonomic composition, indicating that host-specific chemistry, local environmental factors, and regional microbial source pools jointly shape ecological roles. Moreover, the observed patterns of mobile genetic elements and horizontal gene transfer suggest that gene exchange predominantly occurs within microbial lineages, with occasional broader transfers dispersing key functional genes (e.g., those involved in polysaccharide metabolism), which may facilitate microbiome adaptation.

**Supplementary Information:**

The online version contains supplementary material available at 10.1186/s40168-025-02271-y.

## Background

Coniferous forests, which dominate temperate and boreal ecosystems, provide critical ecosystem services such as carbon sequestration [97], regulation of water cycles [11], soil erosion control [99], and habitat for diverse plant and animal species. However, these forests are increasingly under threat from climate change [115]. Current perspectives on forest management often overlook the significance of forest tree microbiomes. These microbiomes comprise ectomycorrhizal (ECM) fungi [47] alongside other fungal and bacterial species adapted to forest vegetation and soil [4]. A better understanding of forest microbiomes is essential due to their role in maintaining forest health, resilience, and biodiversity. A significant portion of this biodiversity resides on the aerial surfaces of plants, also known as the phyllosphere, one of the largest microbial habitats on Earth [75, 124]. Microbes that reside on leaves are vital for nutrient cycling, plant growth, and mitigating both biotic and abiotic stress [75], yet while agricultural and model plant phyllospheres have been extensively studied [66, 72], the phyllospheres of forest trees remain understudied.

The phyllosphere hosts a community of airborne generalists [14] that is shared among plants of different species [104, 124]. Leaf surface microbes are dispersed via aerosols and dust particles, and wind facilitates their movement over short and long distances. While the phyllosphere can act as a passive aerosol sampler [43], persistent microbial communities are maintained through plant–microbe interactions and adaptation to leaf environments [122]. The leaf community of a given plant is influenced by plants growing in the immediate vicinity but also with additional microbial signatures from more distant plant surfaces [81]. Conifer forests provide a vast and enduring reservoir of phyllosphere microbes. Unlike the ephemeral leaves of many agricultural plants, conifer needles offer a long-lived surface for microbial colonization, with individual needles persisting for years to decades [37]. The trees themselves can live for centuries or even millennia, sustaining these microbial communities over extended timescales. Studies of other evergreen plants, such as sagebrush [48], demonstrate that persistent communities of leaf-associated microbes, including at least 20 fungal genera, are maintained over time and are influenced by both weather and leaf age. Similarly, conifers likely provide stable habitats and significant reservoirs for phyllosphere-associated microbial communities, supporting interactions across both nearby and distant habitats and plant ecosystems.

For example, in boreal forests, the composition of the microbial community living on nitrogen‑fixing mosses depends on both the moss species and the surrounding canopy structure [56]. A higher proportion of conifers in the overstory is associated with greater α‑diversity in moss‑associated microbiomes, likely because conifers modify the forest floor environment (e.g., through needle litter, pH, and moisture), creating conditions that support a more diverse microbial community [107, 109]. While microbial dispersal was not directly assessed in [107, 109], conifer-derived microbes, such as those from needle surfaces, may contribute to moss colonization and influence community assembly alongside environmental filtering. Thus, the presence of conifers and the sharing of microbial communities among plant species can enhance microbial diversity and stability in forest ecosystems. Moreover, the ability of conifers to host diverse microbial communities, including nitrifiers that significantly contribute to nitrogen cycling, further underscores their ecological importance as habitats for important phyllosphere communities [44]. Additionally, coniferous trees often endure extreme environmental conditions, such as high UV radiation, low temperatures, and nutrient-poor soils, making them ideal for studying stress tolerance mechanisms in phyllosphere communities. Conifer species exhibit diverse needle characteristics that potentially influence the taxonomic and functional composition of microbial communities on their leaf surfaces. Secondary metabolites like terpenes and phenolic compounds in needles create unique chemical environments [34], selecting for specific microbial taxa that are tolerant of, or potentially capable of metabolizing, these compounds [61].

In parallel, polysaccharide decomposition is another critical microbial function in the phyllosphere with broader implications for forest carbon cycling. Conifer needle surfaces contain complex plant polymers such as cellulose, pectin, and hemicellulose derived from cuticles and cell walls. Among the microbes that inhabit these surfaces, members of the Bacteroidetes phylum are common colonizers [124] and are well equipped to degrade such polymers due to their diverse repertoires of carbohydrate-active enzymes (CAZymes) [6]. Recent work suggests that phyllosphere microbial communities influence ecosystem-level processes, including the “home-field advantage” in litter decomposition, where microbes accelerate decomposition of their host’s own litter relative to foreign litter sources [39]. This advantage could arise because early colonization and metabolism of polysaccharides by phyllosphere organisms creates priority effects, influencing microbial succession and subsequent litter breakdown in soils. However, the prevalence and genomic mechanisms underlying polysaccharide decomposition specifically in conifer phyllosphere microbiomes remain largely unexplored.

Bacterial communities on conifer needles have been implicated in nitrogen fixation and growth promotion [7, 84], both of which contribute to stress resilience. Additional mechanisms by which phyllosphere microbes may enhance conifer stress tolerance include protection against extreme temperatures via heat shock and antifreeze proteins [57], desiccation resistance through biofilm formation or production of extracellular polysaccharides that retain moisture [106], and the synthesis of protective pigments such as carotenoids that absorb or mitigate UV radiation [58]. A recent genome-centered study of switchgrass and miscanthus phyllosphere communities revealed that these microbes harbor genes for osmoprotectants, compatible solutes, and antioxidative enzymes like catalases and peroxidases, which detoxify reactive oxygen species generated under environmental stress [51]. Both conifers and grasses are exposed to similar abiotic stresses, such as drought temperature extremes, and oxidative stress, which can select for analogous stress tolerance functions in their leaf-associated microbiomes [122]. Thus, similar microbial mechanisms may be at work in the conifer phyllosphere. In addition to these adaptations, phyllosphere microbes may also influence trace gas dynamics, including methane oxidation [15], although the extent and mechanisms of methane cycling in conifer needle microbiomes remain poorly understood. Beyond abiotic stress mitigation, phyllosphere microbes, including both bacteria and fungi, play critical roles in defending their host plants against biotic stress. Leaf surface communities can directly suppress pathogens and herbivores through competitive exclusion [75], production of antimicrobial and antiherbivore compounds [85], and induction of systemic resistance. For instance, fungal endophytes in conifer needle tissues have been shown to reduce infection severity by pathogenic fungi and enhance resistance to insect herbivory [3, 22], while bacterial communities further contribute to pathogen suppression by competing for nutrients and space and producing antibiotics, thereby shaping plant community dynamics and improving overall plant fitness [54]. Despite these promising roles, targeted studies on microbial-driven biotic stress tolerance in conifer phyllospheres remain relatively sparse, underscoring the need for further investigation into these complex interactions.

Furthermore, mobile genetic elements (MGEs), including phages, prophages, plasmids and transposons, may facilitate microbial adaptation to environmental stressors and host defenses through horizontal gene transfer (HGT) [65, 112]. Although most research on MGEs has focused on soil and rhizosphere microbiomes, plant associated habitats such as the phyllosphere also harbor MGEs and antibiotic resistance genes (ARGs) that contribute to microbial functional plasticity [52]. Interestingly, large-scale metagenomics comparisons suggest that phyllosphere-associated bacteria may be relatively depleted in MGEs compared to other environments [71], potentially reflecting distinct ecological constraints on horizontal gene transfer in this habitat [10]. Nevertheless, those MGEs that persist may contribute to stress response, chemical defense, or metabolic flexibility, underscoring the need to better characterize their roles in conifer needle microbiomes.

Studies using 16S rRNA-based profiling of bacterial needle communities, including work by Carper et al. [20] and Carrell et al. [21], have shown that the subalpine conifer phyllosphere harbors diverse bacterial populations shaped by a complex interplay between selection and dispersal, with local site factors influencing communities more than host species identity or time across the growing season. However, the functional potential of forest tree phyllosphere communities remains understudied, particularly with respect to key processes such as polysaccharide decomposition, the role of mobile genetic elements (MGEs) in adaptation, and the potential for methane cycling. To our knowledge, no shotgun metagenomics studies of the needle surface exist to date. To address this gap, we sequenced the metagenomes of needle microbiomes from three species of subalpine conifers – limber pine, Engelmann spruce, and Douglas fir – from six sites in the Rocky Mountains, USA, ranging from Glacier National Park, MT (48.70°N) to Rocky Mountain Biological Laboratory, CO (38.96°N) and spanning 1,075 km. We generated 67 metagenomes, where each was assembled and binned into Metagenome Assembled Genomes (MAGs), but we also utilized three large coassemblies specific to each host conifer species, creating a combined, dereplicated set of MAGs that was used to assess overall genome-level abundance, diversity, and functional potential within the conifer needle microbiomes. By generating the first broad‑scale metagenomic survey of conifer‑needle microbiomes spanning six Rocky Mountain sites and three host species, this study establishes a critical baseline for future work on phyllosphere ecology and function, providing an important foundational resource for understanding the genomic diversity and functional potential of these unique phyllosphere communities. Examining the long-lived, aerial surfaces of conifers, key reservoirs for microbial dispersal across landscapes, this work offers insight into microbial connectivity, persistence, and influence beyond the immediate leaf surface, shaping broader forest and ecosystem processes.

## Methods

### Conifer needle sampling and DNA extraction

Conifer needles were collected from each of the three conifer tree species at each of the six different sampling locations. For each tree, approximately 10 g of tissue (a twig with needles) was collected at breast height from the north side of the tree using sterile razor blades and placed into sterile bags. The samples were then placed on ice and shipped overnight to the University of California, Merced. Two 1-g samples were placed in 50 mL tubes, immersed in 15 mL of PBS-S buffer, vortexed for 15 s, and then sonicated for 5 min. Samples were transferred to a new tube and centrifuged for 15 min at 3200 g. The resulting pellet, consisting of needle surface microbes (bacteria and microbial eukaryotes), was resuspended in 1.5 mL of PBS, transferred to a 2 mL tube, centrifuged for 5 min at 10 000 g, and frozen at −80 C until extraction. A CTAB protocol described in Carper et al. [20] was used to extract DNA. This protocol was designed to exclude cells from inside the needle tissue.

### Metagenomic DNA sequencing

Illumina libraries were prepared using standard JGI protocols, i.e. following the protocols described in [12]. Briefly, the Nextera XT kit (Illumina) was used with 2 ng of DNA that was fragmented and adapter ligated. Ligated DNA fragments were PCR amplified and purified using SPRI beads (Beckman Coulter). Library concentrations were determined with quantitative PCR (qPCR) using a LightCycler 480 real-time PCR instrument (Roche). Sequencing was performed on the HiSeq 2500 platform, generating 150 bp paired-end reads.

### Metagenome assembly and annotation

Paired end reads were trimmed and screened according to BBTools documentation [5] and corrected using BFC [73] version r191. Reads without a mate pair were removed. Following trimming and error correction, reads were assembled using metaSPAdes [91]. The filtered read set was mapped to the final assembly where coverage statistics were generated with BBMap version 38.22 using default parameters. Coassemblies were generated for each host tree species (Engelmann spruce, *n* = 18, limber pine, *n* = 31, and douglas fir, *n* = 18) with MetaHipMer2 [50] using default settings. It is worth noting that an attempt was made to create a coassembly of all 67 metagenomes, however, all attempts failed, which prompted us to perform coassemblies of metagenomes within each tree species. Small contigs, less than 500 bp were removed, and coverage information was generated internally in Metahipmer2 during post assembly processing. For individual assemblies, coverage information was generated using BBTools version 38.79 (pileup.sh using default parameters. Functional annotation of the assembled contigs was performed using the IMG annotation pipeline [25] and eggNOG-mapper v2 [17]. CRISPR elements were predicted within IMG via a modified version of CRT-CLI version 1.2 [8]. rRNA genes (5S, 16S, 23S) were identified by comparing the contigs against the Rfam version 13.0 database [86] via cmsearch from the Infernal version 1.1.3 package [87]. Prediction of tRNAs was performed using tRNAscan-SE [23] version 2.0.8. A combination of GeneMarkS-2 version 1.05 [78] and Prodigal version 2.6.3 [53] were used to predict protein-coding genes.

### MAG production and quality control

Metagenome binning was performed using MetaBAT2 [59] with default parameters, using both composition and coverage to generate the MAGs analyzed in this study. All MAGs were quality assessed using CheckM2 [27], and completeness and contamination estimates for each genome are provided in the Supplementary Excel Table. To ensure consistency across both bacterial and eukaryotic MAGs, we adopted the numerical cutoffs from the bacterial minimal information about metagenome-assembled genome (MIMAG) standards [13] rather than specifically assigning HQ/MQ/LQ labels, which were not defined for eukaryotes at the time of publication, including categorization into MIMAG standards [13]. Genes were called and annotated using the Integrated Microbial Genomes (IMG) system at the DOE Joint Genome Institute (JGI) [26]. Furthermore, all genomes were subject to a combined pairwise genomic ANI analysis using fastANI [55] with a 95% identity and 70% alignment threshold to define species-level clusters. Genomes were then clustered into species-level groups using MCL [123] version 14–137 with an inflation parameter of 1.5.

### Phylogenomics of bacterial MAGs

A 56 single copy concatenated marker gene tree was constructed by combining a set of reference genomes spanning the bacterial domain together with the individually assembled and coassembled MAGs. To generate a phylogenetically broad yet non-redundant set of references, we clustered all publicly available IMG isolate bacterial genomes using CD-HIT [76], based on the RNA polymerase gene (rpoB) at 80% sequence identity. This clustering produced a reduced set of reference genomes, roughly unique at the genus to family taxonomic levels (see also Supplementary Fig. 1). The MAGs were dereplicated by grouping them into species-level groups using Mash [96] version 2.0 at a cutoff distance of 0.05, followed by clustering with MCL [123] version 14–137 with an inflation parameter of 1.5. This dereplicated set of MAGs and the reduced set of bacterial reference genomes were passed through the SGTree version 0.0.10 pipeline (https://github.com/NeLLi-team/sgtree). Briefly, this pipeline extracts sets marker genes from the query and reference genomes using hmmsearch [82] version 3.1b2, performs alignments of each marker with MAFFT [60] version v7.490 (2021/Oct/30) using the mafft-linsi option, trims alignments with trimAl version1.4 [18], and removes sites when more than 90% of taxa contain a gap. Due to various levels of MAG completeness, not all MAGs are included in the bacterial phylogeny (Fig. 2A; specifically, MAGs with less than 20% completeness were excluded, resulting in the omission of 10% of MAGs from the tree. Finally, individual protein alignments were concatenated, followed by maximum likelihood tree construction with IQ-TREE [89] multicore version 1.6.1 using the WAG substitution model and ultrafast bootstrap with 1,000 replicates [49]. Trees were visualized with ggtree v3.2.1 [130].

### Phylogenomics of eukaryotic MAGs

To place the eukaryotic MAGs from the conifer needle phyllosphere into a concatenated marker gene tree, we used BUSCO_Phylogenomics (https://github.com/jamiemcg/BUSCO_phylogenomics). This is a pipeline that relies on extracted BUSCO [116] marker genes. To create a reference dataset, we ran BUSCO on the full set of eukaryota_odb10 reference genomes to obtain a BUSCO profile for each reference. We then ran BUSCO on all eukaryotic MAGs with BUSCO completeness scores greater than 50%. These MAGs were defined as eukaryotic based on the identification of Eukaryotic contigs with EukRep [127]. EukCC [113] was then run to further assess quality. The full set of BUSCO generated output consisting of the eukaryota_odb10_reference database and our query MAGs was dereplicated into species clusters at a Mash [96] version 2.0 distance of 0.05. The dereplicated eukaryotic MAGs together with the eukaryotic reference set were then passed to the BUSCO_phylogenomics pipeline, which uses IQ-TREE applying the WAG substitution model and ultrafast bootstrap with 1,000 replicates [49]. Trees were visualized with ggtree v3.2.1 [130].

### MAG abundances

To assess the taxonomic composition of the conifer phyllosphere, we determined the relative abundance of microbial taxa by mapping metagenomic reads to the set of representative MAGs described above. Metagenomic reads from each sample were then mapped to this reference set of dereplicated MAGs using BBSplit from the BBMap suite (v38.90), which allows for unambiguous assignment of reads to the most similar reference genome.

Host-derived MAGs were identified and excluded from microbial community analyses. For community‑level composition, we expressed the number of reads assigned to each microbial MAG as a proportion of all mapped reads within that sample. Taxonomic classifications were assigned to each MAG based on its placement in a reference phylogeny (See Phylogenomics of MAGs above). The resulting abundance data was processed in R (v4.2.0) [103] to visualize taxonomic composition across host plants and sampling sites. Hierarchical clustering of samples was performed using Bray–Curtis dissimilarity on abundance data rarefied to the minimum sample sum (i.e., the lowest total count across samples) using the rrarefy() function from the vegan R package [93]. Dendrograms were constructed to visualize relationships between metagenomes from different host plants and collection sites. Abundance profiles are displayed as stacked bar plots showing the relative abundance of major taxonomic groups, with taxa contributing less than 1% relative abundance grouped into an "Other" category. This approach allowed us to examine the influence of both host plant species and geographical location on microbial community structure in the conifer phyllosphere. Taxa enrichment was determined using DESeq2 [79], a differential abundance testing framework based on the negative binomial distribution that is specifically designed for raw count data. Raw reads assigned to each taxon were used directly for DESeq2 analysis, while filtering out low-abundance taxa that had either fewer than 100 total reads across all samples or fewer than 10 reads in at least 3 samples. For each host species or site, a binary contrast was established comparing the target group against all others. *P*-values were adjusted using the DESeq2 built-in procedures to control for false discovery rate (FDR). For statistical analyses and tabular presentation, taxa were considered significantly enriched or depleted if FDR < 0.01. For visualization purposes (Supplementary Fig. 2), a relaxed threshold of FDR < 0.5 was applied to provide a broader view of potential enrichment patterns. Log2 fold change was calculated directly by DESeq2, representing the log-ratio of normalized counts between target and other groups. The size of points in the supplementary figures represents -log10(FDR), allowing visual distinction between highly significant and marginally significant results.

To assess differences in community composition among samples, we performed non-metric multidimensional scaling (NMDS) and permutational multivariate analysis of variance (PERMANOVA) using the vegan R package [94]. Bray–Curtis dissimilarity matrices were calculated from rarefied abundance tables. NMDS ordinations were generated with the metaMDS function (trymax = 100, k = 2), and stress values were reported [28]. PERMANOVA was conducted using the adonis2 function with 999 permutations to test for significant effects of host species and site on community structure [2]. Ordination plots were visualized with ggplot2 [128], including convex hulls for group separation, biplot arrows for influential taxa, and annotation of PERMANOVA *p*-values and *R*^*2*^ statistics.

### Orthologue clustering

For gene content comparisons, annotations of individual genes were used in combination with gene family clustering using OrthoFinder 2.1.3 [35], and the dereplicated species level MAG collections as input. Only medium quality MAGs and higher were used for orthologue clustering.

### Identification of genes potentially relevant to volatile organic compound degradation

To assess the potential for conifer microbes to degrade common volatile organic compounds (VOCs), we searched the annotations of all MAGs for indicators of common VOC compound degradation such as alpha-pinene, methanol, among others. See Supplemental Excel Table for a full list.

### Identification and characterization of carbohydrate-active enzyme gene families

To identify and classify carbohydrate-active enzyme (CAZyme) gene families, we utilized dbCAN3 [131], a widely used tool for the annotation of CAZymes based on the CAZy database [77]. Predicted protein sequences from all MAGs were screened against the dbCAN3 database using HMMER [100] version 3.3.2. Hits were filtered based on a stringent e-value cutoff (< 1e-15) and coverage threshold (> 35%) to ensure high confidence in CAZyme identification. The outputs from dbCAN3 were further corroborated by cross-referencing annotations generated through the IMG pipeline [26]. To characterize the functional diversity and potential ecological roles of CAZymes, we categorized identified genes into functional groups, including glycoside hydrolases (GHs), glycosyltransferases (GTs), carbohydrate esterases (CEs), polysaccharide lyases (PLs), and auxiliary activities (AAs). Each group was analyzed for their distributions across the MAGs. Substrate utilization profiles were analyzed by mapping CAZyme annotations to their associated substrate types based on the dbCAN3 database. CAZyme families annotated with multiple substrate preferences were counted in all relevant substrate categories, such that each enzyme was included in the normalized count for every substrate it is predicted to target. For each taxonomic group, we calculated the normalized count of substrate-degrading enzymes per genome to account for differences in sampling depth across taxa. The normalized count represents the average number of enzymes targeting each substrate type that would be found in a typical genome from that taxonomic group.

### Detection of mobile genetic elements and their host associations

To identify MGEs, including viruses and plasmids, from both the individual assemblies and coassemblies, contigs were screened with geNomad version 1.1.0 [16] and VirSorter2 version 2.2.4 [45], taking the union of both results as the full set. MGE completeness was assessed with CheckV version 1.0.1 (database version 1.4) [88]. MGEs were then clustered into OTUs using skani [117] with a 90% sequence identity and a 70% alignment fraction. Hosts were assigned to MGEs if they were either binned directly into a MAG or were assigned to a host based on the host prediction tool, iPhoP [110]. To complement the automated prediction of MGEs we also searched for a small set of common phage, conjugative and non-conjugative plasmid and genes within the annotations. The full set of genes used in these searches are available in the Supplemental Excel Table.

### Detection of horizontally transferred genes

Horizontal gene transfer (HGT) was analyzed among all dereplicated MAGs using MetaCHIP [119], a pipeline designed to detect HGT events in microbial genomes by assessing phylogenetic incongruence and taxonomic discrepancies. All dereplicated MAGs were used as input, and the pipeline was run with default parameters to ensure consistency and reproducibility. MetaCHIP begins by identifying candidate HGT genes through sequence similarity searches and taxonomic classification. Genes are flagged as potential HGT candidates if their predicted taxonomy differed significantly from that of the host MAG. To validate these candidates, MetaCHIP constructs phylogenetic analyses, generating gene trees for each candidate and comparing them to the species tree constructed from single-copy marker genes (Fig. 2A). Significant topological discrepancies between the gene trees and the species tree were flagged as evidence of HGT. To reduce false positives, additional filters were applied, including minimum alignment coverage and sequence identity thresholds. Confirmed HGT events were classified into donor-recipient pairs based on taxonomic relationships and the predicted functions of the transferred genes.

## Results & discussion

### Study sites, tree species, and dataset overview

**Fig. 1:**
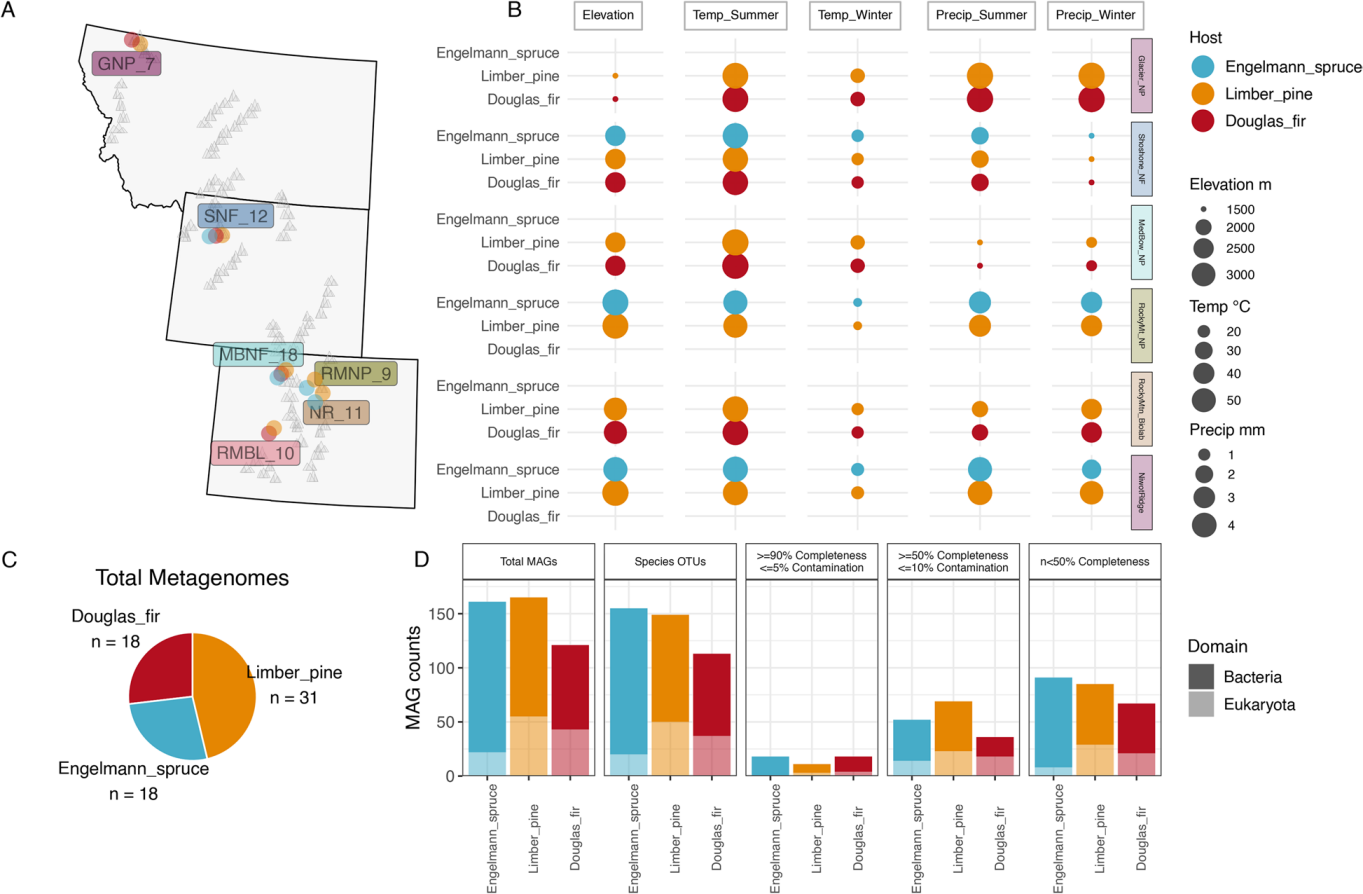
Geographic distribution of sampling sites, their environmental characteristics, and the resulting metagenomic data obtained from three conifer host species. **A** Map showing the sampling sites distributed across the Rocky Mountains, from central Colorado to northern Montana. Site codes (GNP = Glacier National Park, SNF = Shoshone National Forest, MBNF = Medicine Bow National Forest, RMNP = Rocky Mountain National Park, NR = Niwot Ridge, RMBL = Rocky Mountain BioLab) are shown with the total number of samples collected at each location. Circle sizes on the map are uniform and serve as markers for sampling locations. **B** Environmental parameters (elevation, temperature, and precipitation) for each sampling site and host species combination. The size of each circle reflects the underlying metadata value for that site-host combination. Engelmann spruce samples were not collected at GNP, MBNF, and RMBL; Douglas fir samples were not collected at RMNP and NR. **C** Total metagenome distribution by host species, with Limber pine representing the largest sample group (*n* = 31). **D** Counts of bacterial and eukaryotic metagenome-assembled genomes (MAGs) categorized by host species and quality. Species-level OTUs were defined using a 95% average nucleotide identity (ANI) threshold. Completeness and contamination thresholds follow the bacterial MAG criteria of Bowers et al. [13] (“MIMAG” standards [13]). Because MIMAG quality categories (high/medium/low) are currently not defined for eukaryotic MAGs at the time of publication, we use these explicit numerical thresholds for all MAGs rather than the HQ/MQ/LQ labels

This study builds on prior 16S rRNA gene amplicon based research investigating bacterial communities of limber pine and co-occurring conifers across the limber pine species range, spanning sites from California to Colorado and New Mexico to Montana [20]. For the current study, we chose a subset of samples from this broader dataset to apply shotgun metagenomics, allowing deeper insights into microbial community composition and functional potential than previously possible with amplicon sequencing. These samples were collected from six sites spanning the Rocky Mountains, from Glacier National Park, MT in the north to the Rocky Mountain Biological Laboratory (RMBL), CO in the south. At each site, three distinct conifer species, limber pine (*Pinus flexilis*), Douglas fir (*Pseudotsuga menziesii)*, and Engelmann spruce (*Picea engelmannii*), representing distinct genera within the family Pinaceae, were sampled, with minor exceptions: Engelmann spruce was not sampled at Glacier National Park or RMBL. Despite these gaps, each site contained limber pine and Douglas fir samples, ensuring robust coverage across the latitudinal gradient (Fig. 1A). Corresponding climate data [20] from each site (Fig. 1B) highlight environmental gradients present across sites.

Our metagenomic dataset includes 67 conifer needle surface samples collected from individual trees across three conifer species: 31 from limber pine, 18 from Douglas fir, and 18 from Engelmann spruce. Each sample yielded a unique shotgun metagenome, resulting in 67 metagenomes, each with a corresponding individual assembly (Fig. 1C, Supplementary Table 1). Additionally, we generated three large-scale coassemblies, one for each host species, to enhance the recovery of low-coverage sequences [31]. The individual assemblies averaged 342 Mb, while the coassemblies spanned an average total size of 19.3 Gb, offering a valuable resource for exploring both taxonomic and functional diversity within the conifer phyllosphere microbiome.

### MAG extraction, domain-level classification and quality assessment

To further characterize the microbial communities identified in these metagenomes, we extracted metagenome-assembled genomes (MAGs) to analyze the taxonomic and functional diversity within the conifer needle microbiome. From 67 conifer needle metagenomes we recovered a total of 447 MAGs, three of which were very large (average 3.7 Gb), host‐derived MAGs reflecting conifer nuclear genome contamination, and seven insect MAGs, including those from *Ixodes scapularis* (tick, average 23 Mb) and *Copidosoma floridanum* (parasitic wasp, average 66 Mb), alongside 437 microbial genomes. Despite the challenges of assembling eukaryotic genomes from metagenomes [114], the host-derived MAGs provide valuable context for understanding sequence read origins, especially given that conifer genomes are notoriously large (typically 20–30 Gb [92],). The host MAGs underscore the difficulty of assembling highly repetitive eukaryotic genomes from metagenomes, as they averaged only 40.1% completeness by EukCC [113] and 8% by BUSCO [116], with BUSCO’s lower estimate likely reflecting a more accurate representation of assembly gaps. These host-derived MAGs offer useful insights into host sequence contamination, a common challenge in phyllosphere microbiome studies described previously [51]. Our analysis revealed that contamination from host sequences was substantial, with a mean of 90.7% ± 2.0% SEM of mapped reads aligning to contaminant MAGs across samples, highlighting the significant challenge of separating microbial biomass from host material in phyllosphere studies. In contrast, the mean percentage of reads mapping to microbial MAGs (bacterial and eukaryotic combined) was 9.3% ± 2.0% SEM. It is also worth noting that chloroplast MAGs were not recovered, a common result given organellar circularity, lower effective coverage, and high sequence similarity that lead to fragmented or unbinned contigs [125].

**Fig. 2:**
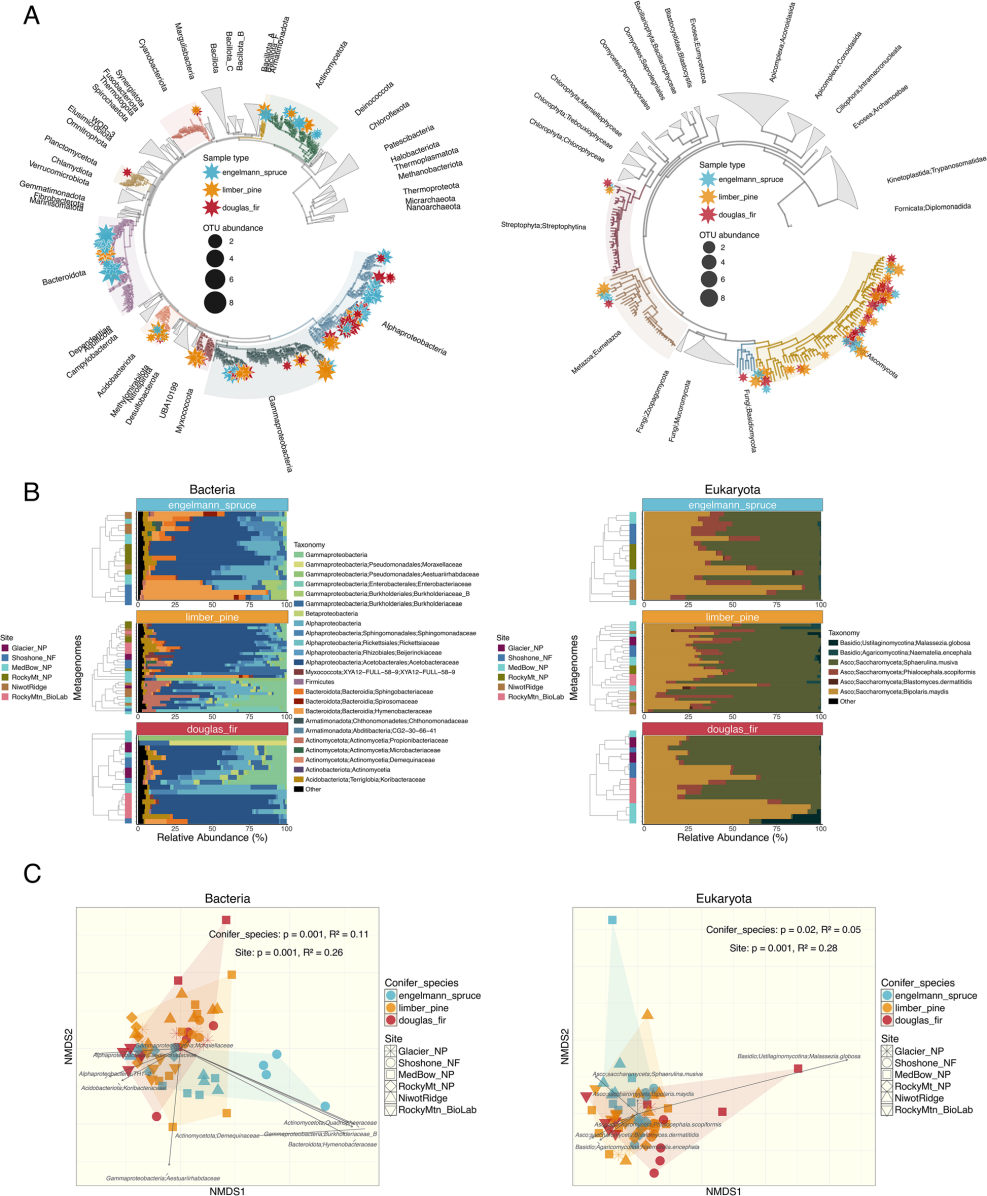
Analysis of bacterial (left-hand panels) and eukaryotic (right-hand panels) metagenomic data: **A** Phylogenetic relationships among recovered MAGs. The bacterial phylogeny is constructed from 56 concatenated marker genes, while the eukaryotic phylogeny is based on the complete set of eukaryotic BUSCO markers. **B** Relative abundances of microbial taxa across different conifer species. Abundances represent the proportion of reads mapped to each MAG, normalized by the total number of reads for the corresponding domain. **C** Community composition visualized through NMDS ordination based on Bray–Curtis dissimilarities (stress values: Bacteria = 0.11, Eukaryota = 0.14). Ordinations use species-level dereplicated sets of MAGs for each domain. Host-associated MAGs, including conifer and insect genomes, are excluded from the Eukaryota relative abundance and ordination panels to focus on microbial taxa

The 447 MAGs (including the 10 contaminant MAGS) were clustered into 417 species‐level MAGs after dereplication, and then partitioned into bacterial and eukaryotic sets. Bacterial MAGs were taxonomically assigned with GTDB-Tk [24] and refined by custom phylogenetic analyses (Fig. 2A). Completeness and contamination estimates, from CheckM2 for bacteria and EukCC for eukaryotes (Supplementary Excel Table), were then used to assign quality based on the numerical cutoffs of the bacterial MIMAG standards [13], applied uniformly since formal HQ/MQ/LQ labels do not yet exist for eukaryotic MAGs. Of 327 bacterial MAGs, 40 met the ≥ 90% completeness ≤ 5% contamination threshold, 102 met ≥ 50% completeness ≤ 10% contamination threshold, and 185 were < 50% complete. Of 117 eukaryotic MAGs, 7 met the ≥ 90% completeness ≤ 5% contamination threshold, 55 met the ≥ 50% completeness ≤ 10% contamination threshold, and 58 were < 50% complete (Fig. 1D).

### Host species and geographic site shape the diversity of conifer-associated microbiomes

The microbial diversity observed in our MAG dataset is consistent with previous findings from amplicon-based studies on limber pine and co-occurring species [20], as well as other investigations into conifer-associated microbiomes, including those on limber pine, lodgepole pine [21], and Monterey pine [1]. While prior studies focused on bacterial communities using amplicon sequencing, our metagenomic approach enables a more comprehensive analysis of both bacterial and fungal communities across multiple hosts and geographic locations.

Bacterial communities in the conifer needle microbiome were dominated by phyllosphere-associated taxa, particularly members of Alphaproteobacteria, Bacteroidota, and Gammaproteobacteria [104]. Less abundant groups included Actinomycetota, Armatimonadota, Acidobacteriota, and Myxococcota. Within Alphaproteobacteria, Acetobacteraceae were the most prominent, with additional representation from Burkholderiaceae, Sphingomonadaceae, and Pseudomonadaceae. Hymenobacteraceae dominated among the Bacteroidota, along with smaller contributions from Sphingobacteraceae and Spirosomaceae (Fig. 2A and B and Supplementary Fig. 1A). Additionally, the presence of Rickettsiaceae in the phyllosphere is intriguing given their typical role as obligate intracellular parasites. Among the eukaryotic MAGs, we recovered bins assigned to Ixodes ticks, which are well‑known reservoirs of Rickettsia endosymbionts [64]. The co‑occurrence of Rickettsiaceae MAGs with the arthropod MAGs suggests that the bacteria originate from endosymbionts harbored by arthropods incidentally collected with the needles, rather than from free‑living rickettsiae or protistan associations. Significant differences in bacterial community composition were detected across host species and sampling sites (PERMANOVA, *p* = 0.0001; Fig. 2C). Community composition was significantly influenced by both site and host species, with site-level variation explaining a slightly larger proportion of the variation in bacterial communities (*R*^*2*^ = 0.26; Fig. 2C) compared to host species (*R*^*2*^ = 0.11; Fig. 2C). Site-level variation likely integrates multiple environmental factors, including climate and microbial dispersal from surrounding habitat, with our prior 16S analysis of a larger set of samples from a wider geographic range showing that land cover exert a stronger influence than climate alone on conifer needle communities [20]. To further quantify these patterns of microbial enrichment across hosts and sites, we performed differential abundance analysis, which revealed 67 significant site and/or host-microbe associations (Table 1, FDR < 0.01).

**Table 1:**
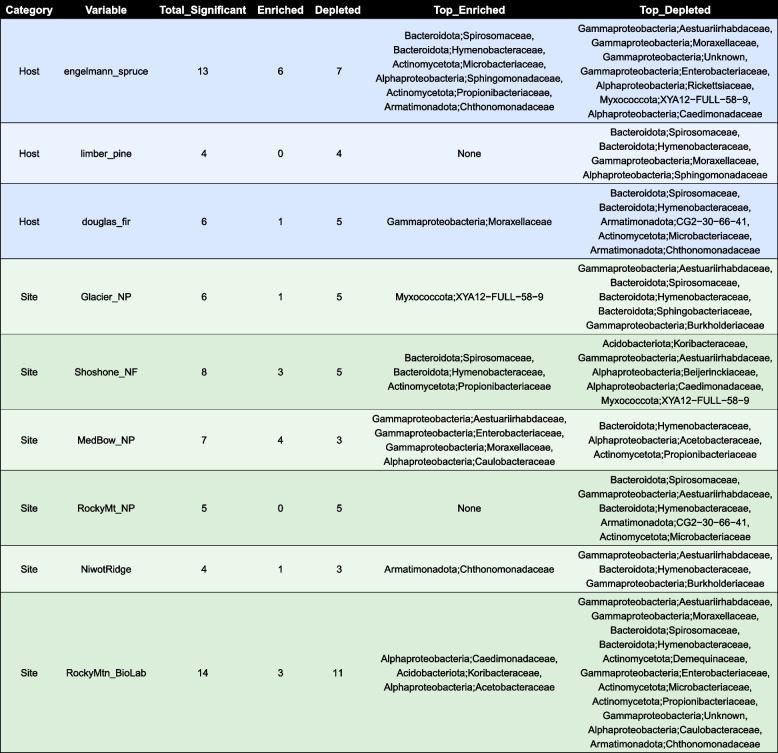
Host and Site-specific enrichment of microbial taxa based on differential abundance analysis. DESeq2 [79] was used to identify significantly enriched or depleted taxa (FDR < 0.01) based on raw read counts. For each host species or site (rows), the table shows the number of significant taxa and their enrichment status, with the most abundant taxa listed. Min. FDR indicates the strongest statistical significance observed for each comparison. Host and site comparisons are distinguished by background colors (blue: hosts,green: sites). All host species and site locations showed significant differential abundance patterns, with a total of 67 significant host-microbe and site-microbe associations detected

Engelmann spruce communities exhibited distinct taxonomic profiles, with significant enrichment of Bacteroidota (Spirosomaceae, Hymenobacteraceae), Actinomycetota (Microbacteriaceae, Propionibacteriaceae), Alphaproteobacteria (Sphingomonadaceae), and Armatimonadota (Chthonomonadaceae) compared to other hosts. The enrichment of Sphingomonadaceae is particularly noteworthy as members of this family are known for their ability to metabolize recalcitrant aromatic compounds [54, 124], potentially providing adaptation to the complex phytochemistry of spruce needles. In contrast, limber pine samples showed significant depletion of several bacterial families including Bacteroidota (Spirosomaceae, Hymenobacteraceae), Gammaproteobacteria (Moraxellaceae) and Alphaproteobacteria (Sphingomonadaceae), suggesting a more selective microbiome. Douglas fir was characterized by elevated levels of Gammaproteobacteria (Moraxellaceae) while showing depletion of several Bacteroidota and Armatimonadota families. The interplay between host and site effects was evident across all sampling locations. At Glacier National Park, Myxococcota (XYA12-FULL-58–9) was significantly enriched, a group rarely reported in phyllosphere studies but known for predatory behavior, including synthesis and secretion of antimicrobial compounds, nutrient cycling via microbial cell lysis, and biofilm modulation through extracellular polymer production [74, 83]. Medicine Bow National Park showed enrichment of several Gammaproteobacteria families (Aestuariirhabdaceae, Enterobacteriaceae, Moraxellaceae) and Alphaproteobacteria (Caulobacteraceae), while Niwot Ridge showed enrichment of Armatimonadota (Chthonomonadaceae). Hymenobacteraceae, which emerged as one of the most prominent bacterial families in our analysis, showed a complex pattern, enriched in Engelmann spruce but depleted in limber pine, Douglas fir, Glacier NP, and Niwot Ridge. This pattern highlights the context-dependent nature of these host-microbe and site-microbe associations [21, 104]. Notably, these taxonomic abundance and enrichment patterns agree with the taxonomic vectors displayed in the ordination plots (Fig. 2C), where the arrows point in the direction of maximum change in abundance for each bacterial family, with their length proportional to the correlation with the ordination axes (*p* < 0.05), revealing the taxa most strongly associated with community differences across sites and hosts.

Fungal diversity, while less taxonomically rich than bacterial diversity, showed comparable site and host-associated variation. Most fungal MAGs were classified within Ascomycota, with smaller contributions from Basidiomycota, consistent with the known dominance of ascomycetes in the phyllosphere [108] (Fig. 2A and B and Supplementary Fig. 1B). Fungal community composition was also significantly influenced by site (PERMANOVA, *p* = 0.001; Fig. 2C), with site-level variation (*R*^*2*^ = 0.28; Fig. 2C) explaining more of the observed differences than host species (*R*^*2*^ = 0.05; Fig. 2C). Several dominant fungal clades included potential pathogens, such as *Bipolaris maydis* and *Sphaerulina musiva*, which are known to cause southern corn leaf blight [121] and poplar cankers [33], respectively. While neither is documented as a conifer pathogen, their presence on needle surfaces likely reflects airborne dispersal, as both produce windborne spores capable of widespread transport. Alternatively, their presence highlights potential gaps in current fungal reference databases, underscoring the need for expanded genomic resources in fungal ecology. Among less dominant fungal taxa, *Phialocephala scopiformis* stood out as a known mutualistic foliar endophyte in conifers. This fungus, which provides protection against spruce budworm [40], was detected across all host species and sites, suggesting it is a generalist capable of colonizing diverse conifer hosts. Its widespread distribution raises intriguing questions about its ecological role and potential protective benefits across varying environmental conditions. Although fungal community composition also varied across hosts and sites, these patterns were less pronounced than those observed for bacterial communities and appeared driven by a few outlier samples. Given the limited phylogenetic diversity of fungal MAGs at broader taxonomic levels (i.e., the dominance of Ascomycota), the remainder of this manuscript focuses on the functional dynamics of bacterial communities.

### Volatile organic compound metabolism: adaptation to conifer chemistry

Trees emit a variety of volatile organic compounds (VOCs), including monoterpenes such as α-pinene, which play important roles in stress adaptation, defense against herbivores and pathogens, and communication between plants [42, 46]. In conifers, VOC emissions are a major component of needle chemistry and may influence the composition and functional capabilities of associated microbial communities. To investigate whether phyllosphere bacteria interact with these host-emitted compounds, we analyzed microbial genes associated with VOC degradation across the needle-associated metagenomes.

**Fig. 3:**
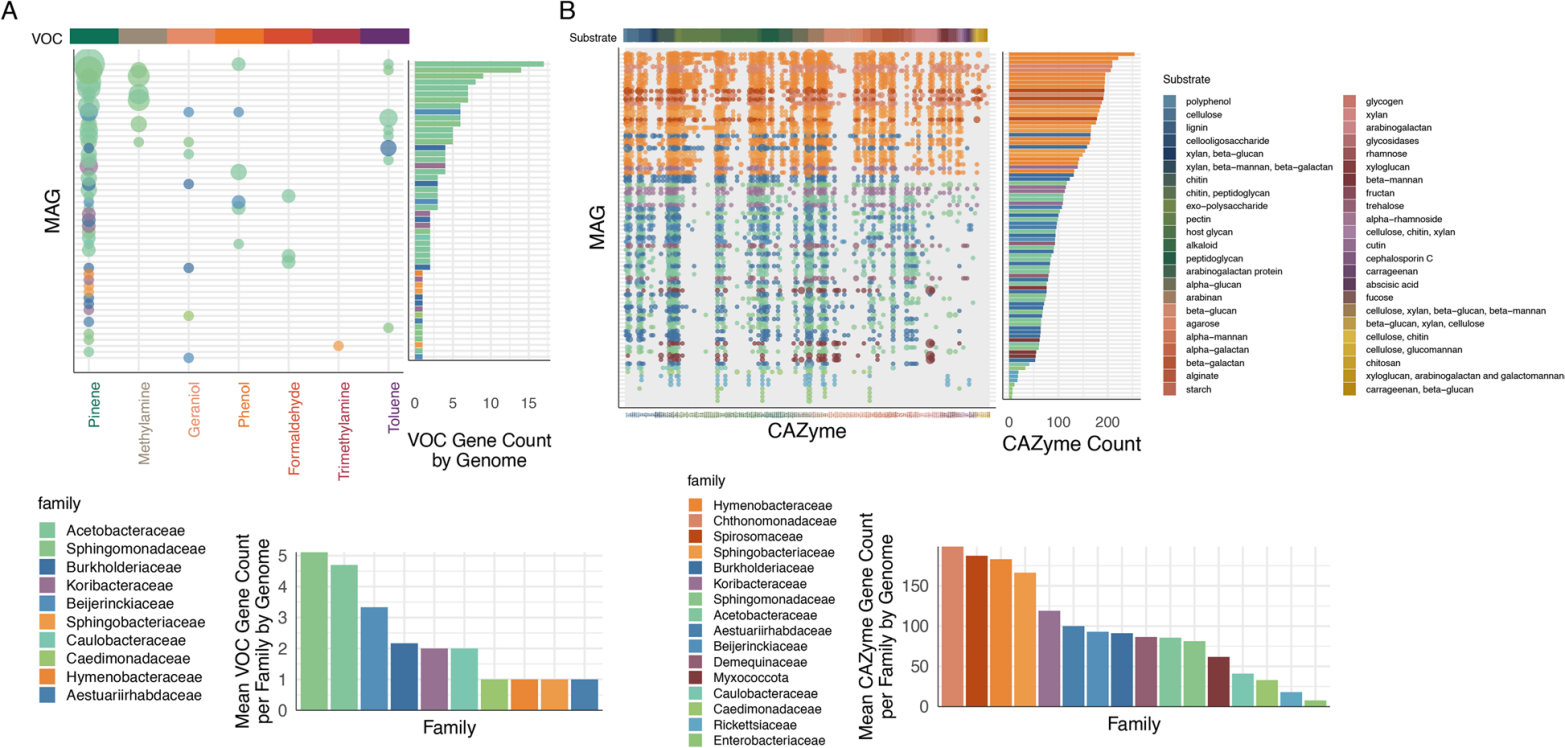
Functional diversity of bacterial communities in the conifer phyllosphere. **A** VOC degradation genes and abundances. Top: Distribution of VOC degradation genes across phyllosphere MAGs, categorized by substrate type. The predominance of α-pinene degradation genes reflects adaptation to conifer-derived monoterpenes. Bottom: Mean VOC-degradation gene count per genome, normalized by the number of genomes per bacterial family, showing Acetobacteraceae and Sphingomonadaceae with the highest abundance of pinene degradation genes. **B** CAZyme diversity and abundances. Top: Total diversity and abundance of CAZyme genes across MAGs, grouped by substrate type. Bottom: Mean CAZyme gene count per genome, normalized by the number of genomes per family-level group, highlighting the exceptional CAZyme repertoire in Bacteroidetes families (Hymenobacteraceae, Sphingobacteriaceae, and Spirosomaceae) and Chthonomonadaceae. The substrate order follows a biochemical gradient from structural plant polysaccharides (cellulose, xylan) to host-derived glycans and specialized substrates

Among VOC-degradation genes, those targeting α-pinene, a monoterpene abundantly produced by conifers as a chemical defense compound, were the most prevalent (Fig. 3A). Within our dataset, pinene degradation genes were primarily associated with bacterial families including Acetobacteraceae, Sphingomonadaceae, and Burkholderiaceae. These taxa likely play key roles in both utilizing these compounds as carbon sources and mitigating their antimicrobial properties, allowing them to thrive on the conifer needle surface. Acetobacteraceae and Sphingomonadaceae showed the highest abundance of genes associated with pinene degradation, suggesting a selective advantage in metabolizing conifer-derived VOCs; a capability that may explain their dominance in the phyllosphere communities we observed across sampling sites. Burkholderiaceae, although less abundant overall, also contributed significantly to VOC degradation potential, underscoring its ecological relevance in specific niches. This pattern of metabolic specialization likely reflects bacterial adaptation to metabolizing the chemical defenses produced by their hosts [75, 124]. The prevalence of pinene-degradation genes suggests that these bacteria not only adapt to the chemical environment of conifer needles but may also contribute to shaping this environment, with implications for tree health, herbivore defense, and climate regulation. Pinene serves multiple ecological roles: it acts as a chemical defense against herbivores and pathogens, deters competing plants, and facilitates communication between trees by signaling threats [62]. In the atmosphere, pinene is highly reactive and plays a crucial role in secondary organic aerosol formation, influencing cloud formation and rainfall patterns [38]. Bacterial communities on needle surfaces may therefore influence pinene's persistence, breakdown, and atmospheric fate.

In contrast, genes targeting other VOCs, such as methanol, geraniol, and phenol, were relatively scarce, indicating limited specialization for these pathways within the sampled bacterial communities. While we did not detect genes associated with methanol oxidation, genes for methane oxidation were identified in known methane-oxidizing taxa, particularly the genus *Methylocystis* within the family Beijerinckiaceae (Supplementary Fig. 3). This finding aligns with previous work reporting methane oxidation potential within conifer-associated microbiomes [101]. However, the sporadic presence of methane oxidation genes in other bacterial families (e.g., Acetobacteraceae and Burkholderiaceae) suggests these taxa likely lack complete functional capabilities for methane oxidation. We also detected near‑complete nitrogen‑fixation (nif) gene sets, most prominently in three of the five MAGs that were again classified as Methylocystis (Beijerinckiaceae), a well‑established diazotroph [29, 120]. Additionally, partial nitrogen fixation pathways were detected in a smaller proportion of MAGs from the families Sphingomonadaceae, Burkholderiaceae, and Aestuariirhabdaceae (e.g., Pseudomonas). Some of these genera may have been responsible for the nitrogenase activity previously detected in limber pine foliage sampled from Niwot Ridge, CO [7, 84], one of the sites represented in this study. Prior work has demonstrated the presence of nitrogen-fixing Sphingomonas isolates in Scots pine needles [7], confirming diazotrophic activity among Sphingomonadaceae in conifer phyllospheres (Supplementary Fig. 3). These findings underscore the potential importance of methane-oxidizing diazotrophs, such as Methylocystis, in coupling carbon and nitrogen cycling within the conifer phyllosphere, particularly under nutrient-limited conditions.

### Carbohydrate-active enzyme distribution: specialized polysaccharide metabolism

Bacterial communities in the conifer phyllosphere exhibited significant functional potential for polysaccharide metabolism, as evidenced by the distribution of carbohydrate-active enzymes (CAZymes) across MAGs (Fig. 3B). Bacteroidetes as a phylum consistently displayed the highest CAZyme functional potential, with the Hymenobacteraceae, Sphingobacteriaceae, and Spirosomaceae. This pattern agrees with the established role of Bacteroidetes as specialists in complex carbohydrate degradation across diverse environments [6]. Interestingly, we also observed two MAGs belonging to *Chthonomonas calidirosea* (phylum Armatimonadota) with exceptionally high CAZyme gene counts. This finding aligns with previous studies of *C. calidirosea* isolates from geothermal environments, which reported extensive carbohydrate-degrading capabilities in this thermophilic bacterium [68].

The CAZyme profiles were broadly similar across bacterial families, with differences primarily observed in the number of gene copies rather than in their presence or absence. For example, Hymenobacteraceae consistently exhibited high CAZyme gene counts, suggesting potential importance in polysaccharide metabolism, a pattern that may be relevant to the enrichment of Hymenobacteraceae within the Engelmann spruce needles at Shoshone NF (Fig. 2B and C, Table 1). We speculate that their CAZyme repertoire may provide a competitive advantage in this specific host-site combination, though experimental validation would be needed to confirm this relationship. The CAZyme repertoire across families targeted a wide range of carbohydrate substrates, including cellulose, xylan, chitin, pectin, and arabinogalactan (Supplementary Fig. 4). The ability to metabolize diverse polysaccharides likely enhances bacterial ecological flexibility, allowing taxa to occupy multiple niches on needle surfaces. By breaking down plant-derived polysaccharides, CAZyme-producing bacteria play a key role in recycling carbon, influencing microbial competition, and modulating the availability of nutrients that shape conifer phyllosphere dynamics. Carbon recycling in the phyllosphere is particularly important because this environment is nutrient-limited and subject to fluctuating abiotic stresses (e.g., desiccation, UV exposure) [75, 124]. Efficient degradation and utilization of both labile (easily degradable, e.g., simple sugars and pectins) and recalcitrant (complex, e.g., cellulose and hemicellulose) substrates can provide a competitive advantage under these conditions [70]. The observed CAZyme patterns suggest that many taxa are equipped to access a range of substrates, which may reflect adaptation to stress by maximizing resource acquisition from available plant polymers. This flexibility likely supports microbial persistence and activity during periods of environmental stress, and may favor the utilization of more labile substrates when available, while also enabling the breakdown of recalcitrant compounds as needed. These processes are critical in nutrient-limited phyllosphere ecosystems, where microbial interactions and competition for resources drive community structure and function [124]. Finally, these CAZyme-mediated functions likely extend beyond the living needle. Upon litterfall, phyllosphere microbes may initiate decomposition, shaping microbial succession and influencing nutrient release. Such early conditioning enhances local decomposition efficiency by favoring well-adapted microbial communities [39], linking phyllosphere metabolism to broader ecosystem carbon cycling.

### Mobile genetic elements and horizontal gene transfer in the conifer phyllosphere

In the harsh and resource-limited environment of the phyllosphere, the ability of microbes to rapidly acquire new traits can be key to survival. Mobile genetic elements (MGEs) and horizontal gene transfer (HGT) are major engines of microbial adaptation, spreading genes for stress tolerance, metabolism, and competition. To explore how gene mobility shapes conifer needle microbiomes, we analyzed the distribution of MGEs and HGT events across community members. Our analysis revealed distinct patterns of genetic exchange, with MGEs primarily mediating lineage-specific transfers and HGT enabling broader cross-taxa gene flow.

**Fig. 4:**
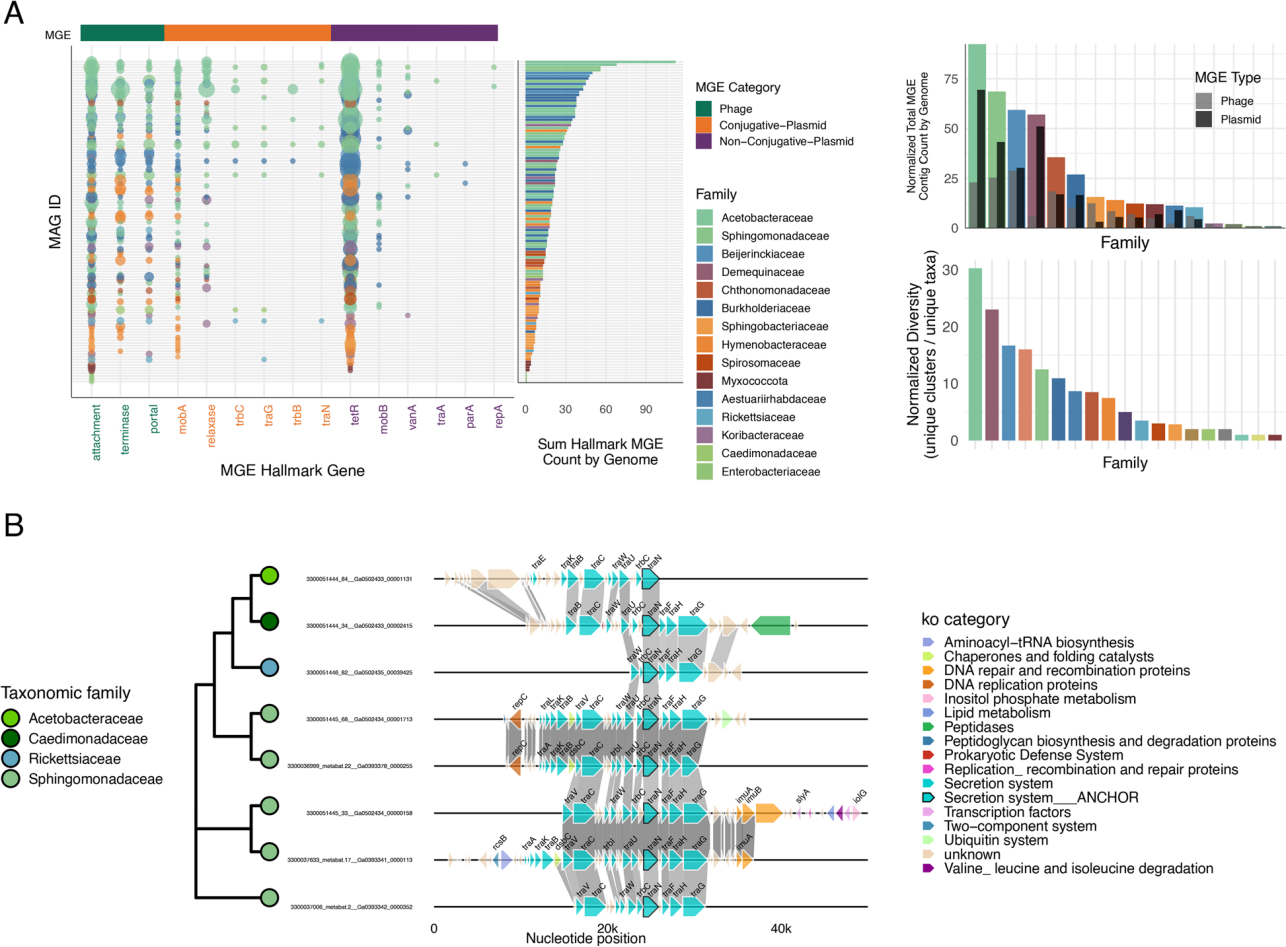
MGE gene counts in phyllosphere MAGs. **A** Left: Distribution and abundance of major MGE hallmark genes across phyllosphere MAGs, showing their prevalence across bacterial lineages and split by predicted phage (green), conjugative plasmid (orange) and non-conjugative plasmid (purple) gene categories. Top right: Average MGE hallmark gene counts per family-level taxonomic group, normalized by the total number of genomes within each family, highlighting enrichment in Alphaproteobacteria families. Bottom right: Mean MGE count per genome, normalized by the total number of genomes within each family. Inset bars show the normalized counts split into phage and plasmid MGE types. **B** Phylogenetic relationships and synteny analysis of conjugative plasmids. The synteny plot illustrates the genomic organization of potential conjugative plasmids across four Alphaproteobacteria families, ordered by the phylogenetic tree of the traN gene, showing conservation of core conjugative machinery genes and variable cargo genes

To investigate the prevalence and diversity of MGEs in the pine needle microbiome, we first scanned MAG annotations for hallmark genes associated with phages, conjugative plasmids, and non-conjugative plasmids. This analysis is summarized in Fig. 4A, which displays the distribution and abundance of these hallmark genes across MAGs and distinct taxonomic families, respectively. To complement the gene-level analysis, we screened for MGE-containing contigs using VirSorter2 [45] and GeNomad [16]. This approach identified 8,956 MGE contigs, including 51 complete phage genomes and 140 high-quality MGEs, with quality assessments performed using CheckV [88]. The results of this contig-level screening are presented as bar charts (Fig. 4A, Right), which summarizes the contig‑level screen for each taxonomic family, the mean number of phage and plasmid associated MGEs per genome and viral diversity, expressed as the number of unique viral OTUs per genome (Fig. 4A, Bottom Right).

Altogether, 26% of the identified MGE contigs could be directly linked to a host MAG through binning, while an additional 0.5% could be linked using iPHoP, which incorporates CRISPR spacer matches and other features for phage-host predictions [111]. The remaining 74% of MGE contigs could not be assigned to a specific host MAG (Supplementary Fig. 5). Nevertheless, nearly all bacterial families hosted at least one MGE, underscoring their ubiquity in the phyllosphere. Based on tetranucleotide ordinations and host-virus interaction networks (Supplementary Fig. 6A and B), we found that the MGEs that could be assigned to a host were predominantly constrained to a single lineage, exhibiting limited host range. For example, MGE sharing was observed between Falsiroseomonas and Commensalibacter, however both of these lineages fall within the Acetobacteraceae family. In contrast, cross-family MGE transfer was rare, with only a few instances detected, such as exchanges involving Methylocystis (Beijerinckiaceae) and either Commensalibacter or Roseomonas_A (Acetobacteraceae). No evidence of widespread cross‑lineage MGE exchange was detected, underscoring the lineage‑specific nature of MGE‑mediated gene flow. MGEs predominantly facilitate gene transfer within closely related taxa, as host specificity is influenced by unique interactions between phage receptor-binding proteins and bacterial surface proteins, which determine the ability of a phage to recognize and infect its host [69, 118]. This pattern aligns with observations in other environments where phage host ranges are typically narrow and constrained by phylogenetic relatedness [32, 63].

In addition to characterizing MGEs at the community level, we identified potential conjugative plasmids within specific bacterial lineages. Using the *traN* gene as an anchor, synteny analysis revealed conserved genomic organization across four Alphaproteobacteria families: Sphingomonadaceae, Acetobacteraceae, Caedimonadaceae, and Rickettsiaceae (Fig. 4B). The traN gene encodes a surface-exposed protein that plays a critical role in mating pair stabilization during conjugative transfer, promoting stable contact between donor and recipient cells [80]. Due to its functional importance and relative conservation among conjugative plasmids, traN serves as a reliable marker for synteny analyses [67]. Conjugative plasmids are a class of self‐replicating mobile genetic elements that carry all of the machinery required for horizontal transfer, namely, a relaxase (e.g., mobA), Type IV secretion system components (e.g., traC, traG, traA, traH), and coupling factors like traN [41]. By shuttling accessory cargo genes (such as imuA, imuB, and dnaE2, which together form a DNA‐damage‐induced mutasome), conjugative plasmids accelerate microbial adaptation under stress and facilitate the spread of novel traits [36, 126]. We also identified toxin-antitoxin systems such as the Type II TA system RelE-RelB [129] and stress-resistance genes like *czcA*, which encodes a cation efflux system conferring heavy metal resistance [90]. Non-conjugative plasmid-associated MGEs revealed notable enrichment of hallmark genes, particularly the TetR-family regulators (Fig. 4A). Although TetR proteins are traditionally associated with antibiotic resistance in clinical settings, they also broadly mediate microbial responses to diverse environmental stressors, including plant-derived metabolites, oxidative stress, and naturally occurring antimicrobial compounds [30, 102]. The frequent occurrence of plasmid-borne TetR regulators in conifer-associated microbial communities underscores their likely ecological importance, independent of anthropogenic antibiotic pressures. Supporting this, George et al. [43] detected tetracycline resistance genes, including TetR regulators, in protected forest conifer samples, far removed from agricultural activity. Taken together, the accessory genes we identified on both conjugative and non-conjugative plasmids, suggests that mobile genetic elements may contribute to stress adaptation in the phyllosphere,a habitat characterized by fluctuating conditions such as UV exposure, desiccation, and nutrient limitation [124].

**Fig. 5:**
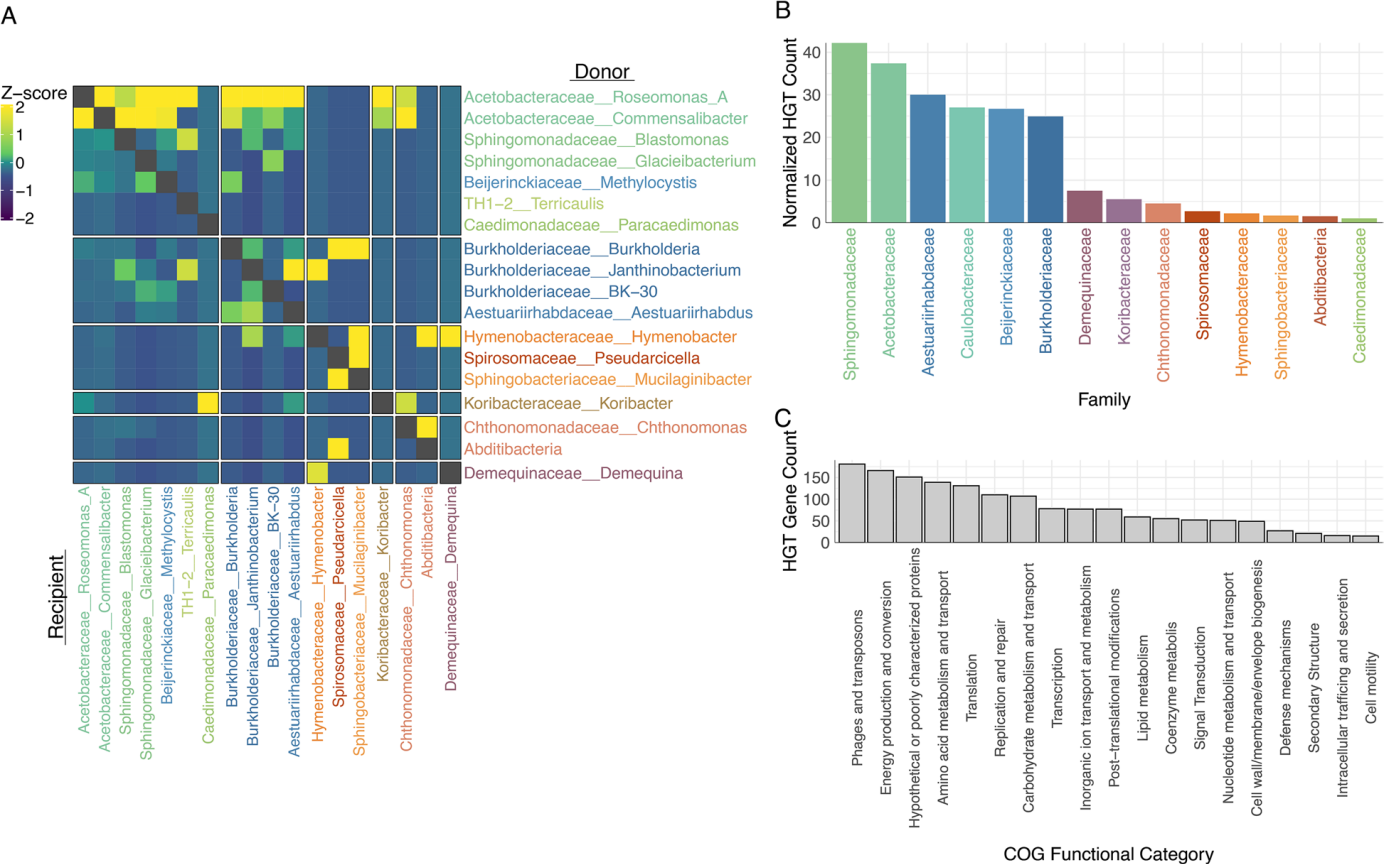
Horizontal gene transfer (HGT) within the conifer needle microbiome. **A** Heatmap displaying the relative abundance of candidate HGT genes, represented as z-scores and grouped by genus, revealing taxonomic hotspots of gene transfer activity particularly in Acetobacteraceae and Sphingomonadaceae. **B** Normalized HGT gene count per genome, grouped by family-level lineage, highlighting variation in HGT frequency across bacterial taxa. **C** Distribution of HGT gene counts across COG functional categories, showing predominance of mobile genetic element functions, such as the Phages and transposons COG category, and hypothetical proteins among horizontally transferred genes

In contrast to the lineage-constrained MGE distributions, horizontal gene transfer (HGT) events displayed greater diversity and frequency, facilitating genetic exchanges across both closely and distantly related taxa. Using MetaChip [119], we identified numerous HGT events spanning diverse bacterial groups within our conifer phyllosphere MAG dataset. The heatmap presented in Fig. 5A highlights the broader scope of HGT, with high abundances of HGT events concentrated in specific taxa, such as Acetobacteraceae and Sphingomonadaceae (Fig. 5B), although the pattern was similar to that of the MGEs, as the total number of HGTs declined with increasing taxonomic distance (Supplemental Fig. 7). This trend suggests that mobile genetic elements (MGEs) play a substantial role in facilitating HGT within certain lineages. However, the widespread occurrence of HGT across diverse taxa suggests that additional exchange mechanisms, beyond MGE activity alone, contribute to horizontal transfer, including recombination and natural DNA uptake [9, 95]. It is likely that some HGT events reflect historical MGE transfers whose direct signatures are no longer detectable [98]. For example, we observed the transfer of CAZyme genes, demonstrating that Acetobacteraceae and Sphingomonadaceae may disperse genes associated with polysaccharide metabolism to other community members (Fig. 5A, C).

The majority of horizontally transferred genes identified in our dataset were associated with phage or transposon-related functions (Fig. 5C) and two of these HGT genes were part of the conjugative plasmid element shown in Fig. 4B. Similar patterns of functional gene transfer have been observed in other plant-associated microbiomes, where genes conferring adaptive advantages in the plant environment show evidence of horizontal transfer [71]. Overall, many of these HGT-associated genes were associated with gene mobility, energy production and conversion, genes of unknown function, and amino acid metabolism and transport, underscoring not only the prevalence of self‐propagating genetic elements but also a functional enrichment that has been previously documented in environmental microbiomes [19, 119].

Together, these processes of both MGE infection and HGT illuminate the complex dynamics of genetic exchange driving microbial adaptation and functional potential in the conifer phyllosphere. The lineage-specific nature of MGEs likely reinforces ecological niche partitioning among bacterial taxa, while broader HGT events may facilitate adaptive gene sharing across more diverse members of the community. These complementary mechanisms potentially contribute to the resilience and functional stability of the phyllosphere microbiome in the face of environmental fluctuations characteristic of the conifer needle habitat.

## Conclusion

This study presents a comprehensive metagenomic analysis of microbial communities associated with conifer needles across a diverse latitudinal gradient in the Rocky Mountains. By leveraging a robust dataset of bacterial MAGs, we uncovered critical insights into the taxonomic and functional diversity of the conifer phyllosphere. Our findings underscore the central role of host-derived compounds, such as volatile organic compounds (VOCs) and complex polysaccharides, in shaping bacterial community structure and driving metabolic adaptations.

Bacterial taxa, particularly members of Acetobacteraceae, Sphingomonadaceae, and Burkholderiaceae, exhibited specialized capabilities for metabolizing dominant monoterpenes like α-pinene, while members of the Bacteroidetes exhibited high potential for breaking down complex carbohydrates such as cellulose and xylan. These metabolic functions underpin their ecological roles, supporting nutrient cycling and microbial community resilience within this unique environment. The spatial patterns observed in community composition, with pronounced host-site interactions exemplified by the Hymenobacteraceae enrichment in Engelmann spruce at Shoshone NF, suggest that host chemistry, local environmental conditions, dispersal rates, and metacommunity structure, all interact to shape the functional potential of needle surface communities.

Predicted MGE contigs were identified as potential mediators of recent and localized gene transfer within closely related bacterial lineages, highlighting their role in short-term evolutionary adaptations. MGEs, including plasmids and phages, were found across nearly all bacterial families, reflecting their ubiquity and contribution to the genomic plasticity of phyllosphere communities. Notably, MGEs were enriched in alpha- and gamma-proteobacteria compared to other taxa, suggesting that these groups may leverage MGEs to enhance their adaptability and ecological success. However, the constrained nature of MGE-mediated transfers suggests that these elements predominantly facilitate gene exchange within taxonomic lineages, supporting functional redundancy and niche specialization in the phyllosphere. In contrast, horizontal gene transfer (HGT) displayed greater diversity and frequency, potentially facilitating the exchange of metabolic genes across both closely and distantly related taxa. HGT emerged as a significant driver of functional diversification, particularly among Acetobacteraceae, which frequently acted as donors of key metabolic traits, including genes involved in polysaccharide metabolism.

Collectively, our findings illuminate how host species and geographic sites influence the complex dynamics that underpin microbial adaptation and resilience within conifer needle environments. By integrating metagenomic, functional, and evolutionary perspectives, this study advances our understanding of conifer needles as significant microbial reservoirs whose microbial diversity and functional potential are closely intertwined with host characteristics and environmental context. These insights lay a foundation for future research into forest phyllosphere microbiomes, emphasizing their ecological roles and contributions to plant health and broader forest ecosystem function.

## Supplementary Information

Supplementary Material 1.

Supplementary Material 2: Table 1. MAG counts by genome type, host species and metagenomic assembly type. Co‑assemblies were generated with MetaHipMer 2 [105], whereas individual metagenomes were assembled with metaSPAdes [91]. Species-level OTUs were defined using a 95% average nucleotide identity (ANI) threshold, for both bacterial and eukaryotic MAGs. The eukaryotic total counts include MAGs derived from host and insect DNA (10 MAGs total). MAG quality is reported in three numerical categories: ≥ 90 % completeness with ≤ 5 % contamination, 50–90 % completeness with ≤ 10 % contamination, and < 50 % completeness, because no community‑endorsed standard yet exists for assigning eukaryotic MAGs to the high‑, medium‑, and low‑quality (HQ/MQ/LQ) categories defined by the bacterial MIMAG guidelines [13].

Supplementary Material 3: Figure 1. Linear representations of bacterial (A, rooted with Archaea) and eukaryotic (B, rooted with Diplomonada) phylogenetic trees. Phyllosphere-derived MAGs are indicated by stars, with size corresponding to the number of MAGs within each species-level group (OTU abundance). Colored clades indicate groups containing at least one phyllosphere MAG. Collapsed clades represent lineages lacking phyllosphere MAGs. Collapsed clades were subsampled to a maximum of 50 tips after phylogenetic inference to highlight relevant clades. The complete phylogenetic analysis was performed using a full dereplicated reference dataset spanning broad diversity across the bacterial and eukaryotic trees. These trees correspond to those depicted in Figure 2, presented here in a linear layout to facilitate more detailed exploration.

Supplementary Material 4: Figure 2: Differential abundance of bacterial taxa across conifer host plants (A) and sampling sites (B) based on DESeq2 analysis. Each panel shows taxa significantly (FDR < 0.01) enriched (green bars) or depleted (red bars) in the target host/site compared to all other samples. Bars represent log2 fold change, with positive values indicating enrichment and negative values indicating depletion in the target condition. The size of black dots corresponds to statistical significance (-log10 of FDR), with larger dots indicating higher significance. Only the top differentially abundant taxa based on fold change magnitude are displayed in each panel.

Supplementary Material 5: Figure 3. Distribution of methane oxidation and nitrogen fixation genes across bacterial taxa. (A) Methane oxidation gene counts by taxon. Each bar represents the total number of KOs (KEGG Orthologs) for each methane oxidation gene (pmoA, pmoB, pmoC, mmoX, mmoY, mmoZ, mmoB, mmoC, mxaF) within each taxonomic group. Taxa are ordered by gene coverage (proportion of genes present) and total KO count. The number of genomes (n) for each taxon is indicated in parentheses. Colors represent different taxonomic groups. (B) Nitrogen fixation gene counts by taxon. Each bar represents the total number of KOs for each nitrogen fixation gene (nifH, nifD, nifK, nifE, nifN, nifB, nifQ, nifF, nifJ, nifA, nifL, anfG, anfD, anfK, nifM, nifS, nifU, nifV, nifW, nifZ, nifX, nifY) within each taxonomic group. Taxa are ordered by gene coverage and total KO count. The number of genomes (n) for each taxon is indicated in parentheses. 

Supplementary Material 6: Figure 4. Distribution of carbohydrate substrate degradation potential across bacterial taxa. The stacked bar plot shows the substrate degradation potential across different bacterial taxonomic groups. Each bar represents a taxonomic group (phylum;family), with Y-axis labels colored by taxonomy. The X-axis shows the normalized count of substrate-degrading enzymes per genome, indicating the average number of enzymes each genome in that taxonomic group possesses for each substrate type. Colors represent different substrate types, ordered from plant cell wall components (blues) through host glycans (greens) to various polysaccharides (reds, oranges, and purples). Taxa are ordered by their total normalized enzyme count, with those at the bottom having the fewest substrate-degrading enzymes and those at the top having the most diverse substrate utilization potential. This visualization reveals substrate specialization patterns across bacterial lineages, highlighting which taxonomic groups are specialized for specific substrate types versus those with broader degradation capabilities.

Supplementary Material 7: Figure 5. Counts of MGEs greater than 5kb, normalized by total assembly size and total number of metagenomes per group. (A) Totals grouped by host and MGE type. (B) Totals grouped by site and MGE type, where shading represents those MGEs observed in a MAG and assigned to a host, those MGEs pulled into a MAG via iPhoP [111], and those MGEs that could not be assigned to a host MAG.

Supplementary Material 8: Figure 6. Mobile genetic element analysis and virus-host network in the phyllosphere microbiome. (A) Principal component analysis (PCA) of tetranucleotide frequencies (TNF) of mobile genetic elements (MGEs) identified from metagenome-assembled genomes (MAGs). The plot illustrates the clustering of MGEs based on nucleotide composition patterns, with axes representing the first two principal components (PC1 and PC2). Each point represents an individual MGE, with colors indicating family level taxonomic identities. (B) Virus-host network visualization depicting predicted relationships between viral populations and their bacterial/archaeal hosts in the phyllosphere. Large colored nodes represent MAG hosts and smaller nodes represent their MGEs including phages, prophages and plasmids.

Supplementary Material 9: Figure 7. Number of cross-taxon Horizontal Gene Transfers (HGTs) across taxonomic ranks. Bar plot showing the total number of inferred cross-taxon horizontal gene transfers (HGTs) identified at five different taxonomic ranks: genus, family, order, class, and phylum. The majority of HGTs occur between closely related taxa (e.g., genus and family), with decreasing numbers observed at higher taxonomic levels.

## Data Availability

Metagenome sequence data is available in NCBI's Sequence Read Archive under BioProject accessions ranging from PRJNA712809 to PRJNA712928. The complete list of 67 metagenome BioSample accessions (SAMN18257876 to SAMN18261528) and their corresponding BioProject IDs are provided in the Supplementary Excel Table. High-quality metagenome-assembled genomes (MAGs) have been deposited at NCBI under 62 BioSample accessions (SAMN48921394 to SAMN48921456), also documented in the Supplementary Excel Table. The assembly accessions will be available upon completion of NCBI processing. Additionally, assemblies, contigs, and genes can be browsed using the JGI IMG platform (https://img.jgi.doe.gov). All MAGs, regardless of quality, have also been deposited in a Figshare repository (https://figshare.com/articles/dataset/Conifer_Phyllosphere_Microbiome_Dataset/29052146), including nucleotide sequences (fna), protein sequences (faa), and Supplemental Data Tables with detailed metadata for each metagenome and MAG, all of which are also included in the manuscript’s Supplementary Excel Table.This comprehensive collection of phyllosphere MAGs and accompanying genomic data serves as a valuable resource for future studies investigating microbial dynamics and virus-host interactions on the phyllosphere, particularly on conifer needles. The authors confirm that all data underlying the findings are fully available without restriction.

## References

[1] Addison S, Armstrong C, Wigley K, Hartley R, Wakelin S. What matters most? Assessment of within-canopy factors influencing the needle microbiome of the model conifer, *Pinus radiata*. Environ Microbiome. 2023;18(1):45. 37254222 10.1186/s40793-023-00507-8 PMC10230745

[2] Anderson MJ. A new method for non-parametric multivariate analysis of variance: non-parametric manova for ecology. Austral Ecol. 2001;26(1):32–46.

[3] Arnold AE, Mejía LC, Kyllo D, Rojas EI, Maynard Z, Robbins N, et al. Fungal endophytes limit pathogen damage in a tropical tree. Proc Natl Acad Sci U S A. 2003;100(26):15649–54. 14671327 10.1073/pnas.2533483100 PMC307622

[4] Baldrian P. Forest microbiome: diversity, complexity and dynamics. FEMS Microbiol Rev. 2017;41(2):109–30. 27856492 10.1093/femsre/fuw040

[5] BBMap. 2022. SourceForge. July 15, 2022. https://sourceforge.net/projects/bbmap/.

[6] Berlemont R, Martiny AC. Phylogenetic distribution of potential cellulases in bacteria. Appl Environ Microbiol. 2013;79(5):1545–54. 23263967 10.1128/AEM.03305-12 PMC3591946

[7] Bizjak T, Sellstedt A, Gratz R, Nordin A. Presence and activity of nitrogen-fixing bacteria in Scots pine needles in a boreal forest: a nitrogen-addition experiment. Tree Physiol. 2023;43(8):1354–64. 37073466 10.1093/treephys/tpad048 PMC10423461

[8] Bland C, Ramsey TL, Sabree F, Lowe M, Brown K, Kyrpides NC, Hugenholtz P. CRISPR Recognition Tool (CRT): a tool for automatic detection of clustered regularly interspaced palindromic repeats. BMC Bioinformatics. 2007;8(1):209. 17577412 10.1186/1471-2105-8-209 PMC1924867

[9] Bobay LM, Ochman H. 2017. Biological species are universal across life’s domains. Genome Biol Evol. 2017. https://academic.oup.com/gbe/article/9/3/491/2982379. 10.1093/gbe/evx026 PMC5381558 28186559

[10] Bograd A, Oppenheimer-Shaanan Y, Levy A. Plasmids, prophages, and defense systems are depleted from plant microbiota genomes. Genome Biol. 2025;26(1):163. 40500753 10.1186/s13059-025-03641-3 PMC12153167

[11] Bonan GB. Forests and climate change: forcings, feedbacks, and the climate benefits of forests. Science. 2008;320(5882):1444–9. 18556546 10.1126/science.1155121

[12] Bowers RM, Clum A, Tice H, Lim J, Singh K, Ciobanu D, et al. Impact of library preparation protocols and template quantity on the metagenomic reconstruction of a mock microbial community. BMC Genomics. 2015;16(1):856. 26496746 10.1186/s12864-015-2063-6 PMC4619416

[13] Bowers RM, Kyrpides NC, Stepanauskas R, Harmon-Smith M, Doud D, Reddy TBK, et al. Minimum information about a single amplified genome (MISAG) and a metagenome-assembled genome (MIMAG) of bacteria and archaea. Nat Biotechnol. 2017;35(8):725–31. 28787424 10.1038/nbt.3893 PMC6436528

[14] Bowers RM, Sullivan AP, Costello EK, Collett JL, Knight R, Fierer N. Sources of bacteria in outdoor air across cities in the Midwestern United States. Appl Environ Microbiol. 2011;77(18):6350–6. 21803902 10.1128/AEM.05498-11 PMC3187178

[15] Bringel F, Couée I. Pivotal Roles of Phyllosphere Microorganisms at the Interface between Plant Functioning and Atmospheric Trace Gas Dynamics. Front Microbiol. 2015;6(May):486. 26052316 10.3389/fmicb.2015.00486 PMC4440916

[16] Camargo AP, Roux S, Schulz F, Babinski M, Xu Y, Hu B, et al. Identification of mobile genetic elements with geNomad. Nat Biotechnol. 2023. 10.1038/s41587-023-01953-y. 37735266 10.1038/s41587-023-01953-y PMC11324519

[17] Cantalapiedra CP, Hernández-Plaza A, Letunic I, Bork P, Huerta-Cepas J. EggNOG-Mapper v2: Functional annotation, orthology assignments, and domain prediction at the metagenomic scale. Mol Biol Evol. 2021;38(12):5825–9. 34597405 10.1093/molbev/msab293 PMC8662613

[18] Capella-Gutiérrez S, Silla-Martínez JM, Gabaldón T. trimAl: a tool for automated alignment trimming in large-scale phylogenetic analyses. Bioinformatics. 2009;25(15):1972. 19505945 10.1093/bioinformatics/btp348 PMC2712344

[19] Caro-Quintero A, Konstantinidis KT. Inter-Phylum HGT Has Shaped the Metabolism of Many Mesophilic and Anaerobic Bacteria. ISME J. 2015;9(4):958–67. 25314320 10.1038/ismej.2014.193 PMC4817696

[20] Carper DL, Lawrence TJ, Quiroz D, Kueppers LM, Frank AC. Needle Bacterial Community Structure across the Species Range of Limber Pine. ISME Commun. 2024;4(1):ycae062. 38800125 10.1093/ismeco/ycae062 PMC11128189

[21] Carrell AA, Carper DL, Frank AC. Subalpine Conifers in Different Geographical Locations Host Highly Similar Foliar Bacterial Endophyte Communities. FEMS Microbiol Ecol. 2016;92(8). 10.1093/femsec/fiw124. 10.1093/femsec/fiw124 27267931

[22] Carroll G. Fungal endophytes in stems and leaves: from latent pathogen to mutualistic symbiont. Ecology. 1988;69(1):2–9.

[23] Chan PP, Lowe TM. tRNAscan-SE: Searching for tRNA Genes in Genomic Sequences. In Gene Prediction: Methods and Protocols, edited by Martin Kollmar, 2019:1–14. New York, NY: Springer New York. 10.1007/978-1-4939-9173-0_1 PMC6768409 31020551

[24] Chaumeil PA, Mussig AJ, Hugenholtz P, Parks DH. GTDB-Tk: a toolkit to classify genomes with the genome taxonomy database. Bioinformatics. 2020;36(6):1925–7. 10.1093/bioinformatics/btz848 PMC7703759 31730192

[25] Chen IMA, Chu K, Palaniappan K, Ratner A, Huang J, Huntemann M, Hajek P, et al. The IMG/M data management and analysis system v.6.0: new tools and advanced capabilities. Nucleic Acids Res. 2021;49(D1):D751–63. 10.1093/nar/gkaa939 PMC7778900 33119741

[26] Chen IMA, Chu K, Palaniappan K, Ratner A, Huang J, Huntemann M, Hajek P, et al. The IMG/M data management and analysis system v.7: content updates and new features. Nucleic Acids Res. 2023;51(D1):D723–32. 10.1093/nar/gkac976 PMC9825475 36382399

[27] Chklovski A, Parks DH, Woodcroft BJ, Tyson GW. CheckM2: a rapid, scalable and accurate tool for assessing microbial genome quality using machine learning. Nat Methods. 2023;20(8):1203–12. 37500759 10.1038/s41592-023-01940-w

[28] Clarke KR. Non-parametric multivariate analyses of changes in community structure. Aust J Ecol. 1993;18(1):117–43.

[29] Cui J, Zhang M, Chen L, Zhang S, Luo Y, Cao W, et al. Methanotrophs Contribute to Nitrogen Fixation in Emergent Macrophytes. Front Microbiol. 2022;13(April):851424. 35479617 10.3389/fmicb.2022.851424 PMC9036440

[30] Cuthbertson L, Nodwell JR. The TetR Family of Regulators. Microbiol Mol Biol Rev. 2013;77(3):440–75. 24006471 10.1128/MMBR.00018-13 PMC3811609

[31] Delgado LF, Andersson AF. Evaluating metagenomic assembly approaches for biome-specific gene catalogues. Microbiome. 2022;10(1):72. 35524337 10.1186/s40168-022-01259-2 PMC9074274

[32] Díaz-Muñoz SL. “Viral Coinfection Is Shaped by Host Ecology and Virus-Virus Interactions across Diverse Microbial Taxa and Environments.” Virus Evol. 2017;3(1):vex011. 28469939 10.1093/ve/vex011 PMC5407056

[33] Dove NC, Carrell AA, Engle NL, Klingeman DM, Rodriguez M, Wahl T, Tschaplinski TJ, Muchero W, Schadt CW, Cregger MA. Relationships between Sphaerulina musiva infection and the Populus microbiome and metabolome. mSystems. 2022;7(4):e0012022. 10.1128/msystems.00120-22 PMC9426494 35862808

[34] Dudareva N, Pichersky E, Gershenzon J. Biochemistry of plant volatiles. Plant Physiol. 2004;135(4):1893–902. 15326281 10.1104/pp.104.049981 PMC520761

[35] Emms DM, Kelly S. Orthofinder: solving fundamental biases in whole genome comparisons dramatically improves orthogroup inference accuracy. Genome Biol. 2015;16(1):157. 26243257 10.1186/s13059-015-0721-2 PMC4531804

[36] Erill I, Campoy S, Mazon G, Barbé J. Dispersal and regulation of an adaptive mutagenesis cassette in the bacteria domain. Nucleic Acids Res. 2006;34(1):66–77. 16407325 10.1093/nar/gkj412 PMC1326238

[37] Ewers FW, Schmid R. Longevity of needle fascicles of *Pinus longaeva* (bristlecone pine) and other North American pines. Oecologia. 1981;51(1):107–15. 28310317 10.1007/BF00344660

[38] Faiola CL, Buchholz A, Kari E, Yli-Pirilä P, Holopainen JK, Kivimäenpää M, et al. Terpene Composition Complexity Controls Secondary Organic Aerosol Yields from Scots Pine Volatile Emissions. Sci Rep. 2018;8(1):1–13. 29445182 10.1038/s41598-018-21045-1 PMC5813208

[39] Fanin N, Lin D, Freschet GT, Keiser AD, Augusto L, Wardle DA, et al. “Home-Field Advantage of Litter Decomposition: From the Phyllosphere to the Soil.” New Phytol. 2021;231(4):1353–8. 34008201 10.1111/nph.17475

[40] Frasz SL, Walker AK, Nsiama TK, Adams GW, Miller JD. “Distribution of the Foliar Fungal Endophyte Phialocephala Scopiformis and Its Toxin in the Crown of a Mature White Spruce Tree as Revealed by Chemical and qPCR Analyses.” Can J Forest Res. 2014;44(9):1138–43.

[41] Frost LS, Leplae R, Summers AO, Toussaint A. Mobile genetic elements: the agents of open source evolution. Nat Rev Microbiol. 2005;3(9):722–32. 16138100 10.1038/nrmicro1235

[42] Garzoli S, Masci VL, Caradonna V, Tiezzi A, Giacomello P, Ovidi E. Liquid and vapor phase of four conifer-derived essential oils: comparison of chemical compositions and antimicrobial and antioxidant properties. Pharmaceuticals (Basel, Switzerland). 2021;14(2):134. 33567501 10.3390/ph14020134 PMC7914598

[43] George PBL, Leclerc S, Turgeon N, Veillette M, Duchaine C. “Conifer Needle Phyllosphere as a Potential Passive Monitor of Bioaerosolised Antibiotic Resistance Genes.” Antibiotics. 2022;11(7):907. 35884161 10.3390/antibiotics11070907 PMC9312085

[44] Guerrieri R, Cáliz J, Mattana S, Barceló A, Candela M, Elustondo D, et al. Substantial contribution of tree canopy nitrifiers to nitrogen fluxes in European forests. Nat Geosci. 2024;17(2):130–6.

[45] Guo J, Bolduc B, Zayed AA, Varsani A, Dominguez-Huerta G, Delmont TO, et al. VirSorter2: a multi-classifier, expert-guided approach to detect diverse DNA and RNA viruses. Microbiome. 2021;9(1):37. 33522966 10.1186/s40168-020-00990-y PMC7852108

[46] Hakola H, Tarvainen V, Laurila T, Hiltunen V, Hellén H, Keronen P. “Seasonal Variation of VOC Concentrations above a Boreal Coniferous Forest.” Atmos Environ. 2003;37(12):1623–34.

[47] Heijden MGAVD, Martin FM, Selosse M-A, Sanders IR. “Mycorrhizal Ecology and Evolution: The Past, the Present, and the Future.” New Phytol. 2015;205(4):1406–23. 25639293 10.1111/nph.13288

[48] Heil JA, Simler-Williamson A, Striluk ML, Trawick D, Capezza R, DeFehr C, et al. Weather and leaf age separately contribute to temporal shifts in phyllosphere fungal community structure in sagebrush. Ecosphere. 2025;16(6):e70295.

[49] Hoang DT, Chernomor O, von Haeseler A, Minh BQ, Vinh LS. UFboot2: improving the ultrafast bootstrap approximation. Mol Biol Evol. 2018;35(2):518–22. 29077904 10.1093/molbev/msx281 PMC5850222

[50] Hofmeyr S, Egan R, Georganas E, Copeland AC, Riley R, Clum A, Eloe-Fadrosh E, et al. Terabase-scale metagenome coassembly with MetaHipMer. Sci Rep. 2020;10(1). 10.1038/s41598-020-67416-5. 10.1038/s41598-020-67416-5 PMC7329831 32612216

[51] Howe A, Stopnisek N, Dooley SK, Yang F, Grady KL, Shade A. Seasonal activities of the phyllosphere microbiome of perennial crops. Nat Commun. 2023;14(1):1039. 36823152 10.1038/s41467-023-36515-y PMC9950430

[52] Huang X-R, Neilson R, Yang L-Y, Deng J-J, Shu-Yi-Dan Zhou Hu, Li Y-G, et al. Urban Greenspace Types Influence the Microbial Community Assembly and Antibiotic Resistome More in the Phyllosphere than in the Soil. Chemosphere. 2023;338(139533):139533. 37459932 10.1016/j.chemosphere.2023.139533

[53] Hyatt D, Chen G-L, Locascio PF, Land ML, Larimer FW, Hauser LJ. “Prodigal: Prokaryotic Gene Recognition and Translation Initiation Site Identification.” BMC Bioinformatics. 2010;11(March):119. 20211023 10.1186/1471-2105-11-119 PMC2848648

[54] Innerebner G, Knief C, Vorholt JA. Protection of *Arabidopsis thaliana* against leaf-pathogenic *Pseudomonas syringae* by *Sphingomonas* strains in a controlled model system. Appl Environ Microbiol. 2011;77(10):3202–10. 21421777 10.1128/AEM.00133-11 PMC3126462

[55] Jain C, Rodriguez-R LM, Phillippy AM, Konstantinidis KT, Aluru S. High throughput ANI analysis of 90K prokaryotic genomes reveals clear species boundaries. Nat Commun. 2018;9(1):5114. 30504855 10.1038/s41467-018-07641-9 PMC6269478

[56] Jean M, Holland-Moritz H, Melvin AM, Johnstone JF, Mack MC. “Experimental Assessment of Tree Canopy and Leaf Litter Controls on the Microbiome and Nitrogen Fixation Rates of Two Boreal Mosses.” New Phytol. 2020;227(5):1335–49. 32299141 10.1111/nph.16611

[57] Jia Z, Davies PL. Antifreeze proteins: an unusual receptor-ligand interaction. Trends Biochem Sci. 2002;27(2):101–6. 11852248 10.1016/s0968-0004(01)02028-x

[58] Joshi K, Kumar P, Kataria R. “Microbial Carotenoid Production and Their Potential Applications as Antioxidants: A Current Update.” Process Biochemistry (Barking, London, England). 2023;128(May):190–205.

[59] Kang DD, Li F, Kirton E, Thomas A, Egan R, An H, et al. “MetaBAT 2: An Adaptive Binning Algorithm for Robust and Efficient Genome Reconstruction from Metagenome Assemblies.” PeerJ. 2019;7(July):e7359. 31388474 10.7717/peerj.7359 PMC6662567

[60] Katoh K, Standley DM. MAFFT multiple sequence alignment software version 7: improvements in performance and usability. Mol Biol Evol. 2013;30(4):772. 23329690 10.1093/molbev/mst010 PMC3603318

[61] Kembel SW, O’Connor TK, Arnold HK, Hubbell SP, Wright SJ, Green JL. “Relationships between Phyllosphere Bacterial Communities and Plant Functional Traits in a Neotropical Forest.” Proc Natl Acad Sci U S A. 2014;111(38):13715–20. 25225376 10.1073/pnas.1216057111 PMC4183302

[62] Kopaczyk JM, Warguła J, Jelonek T. The Variability of Terpenes in Conifers under Developmental and Environmental Stimuli. Environ Exp Bot. 2020;180(104197):104197.

[63] Koskella B, Meaden S. Understanding bacteriophage specificity in natural microbial communities. Viruses. 2013;5(3):806–23. 23478639 10.3390/v5030806 PMC3705297

[64] Kurtti TJ, Felsheim RF, Burkhardt NY, Oliver JD, Heu CC, Munderloh UG. “Rickettsia Buchneri Sp. Nov., a Rickettsial Endosymbiont of the Blacklegged Tick Ixodes Scapularis.” Int J Syst Evol Microbiol. 2015;65(Pt 3):965–70. 25563918 10.1099/ijs.0.000047 PMC4365292

[65] Ku Y-S, Wang Z, Duan S, Lam H-M. Rhizospheric communication through mobile genetic element transfers for the regulation of microbe-plant interactions. Biology. 2021;10(6):477. 34071379 10.3390/biology10060477 PMC8227670

[66] Laforest-Lapointe I, Messier C, Kembel SW. “Host Species Identity, Site and Time Drive Temperate Tree Phyllosphere Bacterial Community Structure.” Microbiome. 2016;4(1):1–10. 27316353 10.1186/s40168-016-0174-1 PMC4912770

[67] Lawley TD, Klimke WA, Gubbins MJ, Frost LS. F factor conjugation is a true type IV secretion system. FEMS Microbiol Lett. 2003;224(1):1–15. 12855161 10.1016/S0378-1097(03)00430-0

[68] Lee K-Y, Morgan XC, Dunfield PF, Tamas I, McDonald IR, Stott MB. Genomic analysis of *Chthonomonas calidirosea*, the first sequenced isolate of the phylum Armatimonadetes. ISME J. 2014;8(7):1522–33. 24477196 10.1038/ismej.2013.251 PMC4069393

[69] Le S, He X, Tan Y, Huang G, Zhang L, Lux R, et al. Mapping the tail fiber as the receptor binding protein responsible for differential host specificity of *Pseudomonas aeruginosa* bacteriophages PaP1 and JG004. PLoS ONE. 2013;8(7):e68562. 23874674 10.1371/journal.pone.0068562 PMC3706319

[70] Leveau JH, Lindow SE. “Appetite of an Epiphyte: Quantitative Monitoring of Bacterial Sugar Consumption in the Phyllosphere.” Proc Natl Acad Sci U S A. 2001;98(6):3446–53. 11248098 10.1073/pnas.061629598 PMC30673

[71] Levy A, Gonzalez Salas I, Mittelviefhaus M, Clingenpeel S, Herrera Paredes S, Miao J, Wang K, et al. Genomic features of bacterial adaptation to plants. Nat Genet. 2017;50(1):138–50. 10.1038/s41588-017-0012-9 PMC5957079 29255260

[72] Li F, Zi H, Sonne C, Li X. “Microbiome Sustains Forest Ecosystem Functions across Hierarchical Scales.” Eco-Environment & Health. 2023;2(1):24–31. 38074452 10.1016/j.eehl.2023.03.001 PMC10702926

[73] Li H. BFC: correcting Illumina sequencing errors. Bioinformatics. 2015;31(17):2885–7. 25953801 10.1093/bioinformatics/btv290 PMC4635656

[74] Li M, Hong L, Ye W, Wang Z, Shen H. Phyllosphere bacterial and fungal communities vary with host species identity, plant traits and seasonality in a subtropical forest. Environ Microbiome. 2022;17(1):29. 35681245 10.1186/s40793-022-00423-3 PMC9185928

[75] Lindow SE, Brandl MT. Microbiology of the phyllosphere. Appl Environ Microbiol. 2003;69(4):1875. 12676659 10.1128/AEM.69.4.1875-1883.2003 PMC154815

[76] Li W, Godzik A. Cd-hit: a fast program for clustering and comparing large sets of protein or nucleotide sequences. Bioinformatics. 2006;22(13):1658–9. 16731699 10.1093/bioinformatics/btl158

[77] Lombard V, Golaconda Ramulu H, Drula E, Coutinho PM, Henrissat B. The Carbohydrate-Active Enzymes Database (CAZy) in 2013. Nucleic Acids Res. 2014;42(Database issue):D490–95. 10.1093/nar/gkt1178 PMC3965031 24270786

[78] Lomsadze A, Gemayel K, Tang S, Borodovsky M. Modeling leaderless transcription and atypical genes results in more accurate gene prediction in prokaryotes. Genome Res. 2018;28(7):1079–89. 29773659 10.1101/gr.230615.117 PMC6028130

[79] Love MI, Huber W, Anders S. Moderated estimation of fold change and dispersion for RNA-seq data with DESeq2. Genome Biol. 2014;15(12):550. 25516281 10.1186/s13059-014-0550-8 PMC4302049

[80] Low WW, Wong JLC, Beltran LC, Seddon C, David S, Kwong H-S, et al. Mating pair stabilization mediates bacterial conjugation species specificity. Nat Microbiol. 2022;7(7):1016–27. 35697796 10.1038/s41564-022-01146-4 PMC9246713

[81] Meyer KM, Porch R, Muscettola IE, Vasconcelos ALS, Sherman JK, Metcalf CJE, et al. Plant neighborhood shapes diversity and reduces interspecific variation of the phyllosphere microbiome. ISME J. 2022. 10.1038/s41396-021-01184-6. 35022514 10.1038/s41396-021-01184-6 PMC9038669

[82] Mistry J, Finn RD, Eddy SR, Bateman A, Punta M. Challenges in homology search: HMMER3 and convergent evolution of coiled-coil regions. Nucleic Acids Res. 2013;41(12):e121. 23598997 10.1093/nar/gkt263 PMC3695513

[83] Mohr KI, Moradi A, Glaeser SP, Kämpfer P, Gemperlein K, Nübel U, et al. *Nannocystis konarekensis* Sp. Nov., a novel myxobacterium from an Iranian desert. Int J Syst Evol Microbiol. 2018;68(3):721–9. 29458458 10.1099/ijsem.0.002569

[84] Moyes AB, Kueppers LM, Pett-Ridge J, Carper DL, Vandehey N, O’Neil J, Frank AC. Evidence for Foliar Endophytic Nitrogen Fixation in a Widely Distributed Subalpine Conifer. New Phytol. 2016;210(2). 10.1111/nph.13850. 10.1111/nph.13850 27000956

[85] Müller DB, Vogel C, Bai Y, Vorholt JA. The plant microbiota: systems-level insights and perspectives. Annu Rev Genet. 2016;50(1):211–34. 27648643 10.1146/annurev-genet-120215-034952

[86] Nawrocki EP, Burge SW, Bateman A, Daub J, Eberhardt RY, Eddy SR, Floden EW, et al. Rfam 12.0: Updates to the RNA Families Database. Nucleic Acids Res. 2015;43 (Database issue):D130. 10.1093/nar/gku1063 PMC4383904 25392425

[87] Nawrocki EP, Eddy SR. Infernal 1.1: 100-fold faster RNA homology searches. Bioinformatics. 2013;29(22):2933. 24008419 10.1093/bioinformatics/btt509 PMC3810854

[88] Nayfach S, Camargo AP, Schulz F, Eloe-Fadrosh E, Roux S, Kyrpides NC. CheckV Assesses the Quality and Completeness of Metagenome-Assembled Viral Genomes. Nat Biotechnol. 2020;39(5):578–85. 10.1038/s41587-020-00774-7 PMC8116208 33349699

[89] Nguyen L-T, Schmidt HA, von Haeseler A, Minh BQ. Iq-tree: a fast and effective stochastic algorithm for estimating maximum-likelihood phylogenies. Mol Biol Evol. 2015;32(1):268–74. 25371430 10.1093/molbev/msu300 PMC4271533

[90] Nies DH. The cobalt, zinc, and cadmium efflux system CzcABC from *Alcaligenes eutrophus* functions as a cation-proton antiporter in *Escherichia coli*. J Bacteriol. 1995;177(10):2707–12. 7751279 10.1128/jb.177.10.2707-2712.1995 PMC176940

[91] Nurk S, Meleshko D, Korobeynikov A, Pevzner PA. metaSPAdes: a new versatile metagenomic assembler. Genome Res. 2017;27(5):824–34. 10.1101/gr.213959.116. Epub 2017 Mar 15. PMID: 28298430; PMCID: PMC5411777. 10.1101/gr.213959.116 PMC5411777 28298430

[92] Nystedt B, Street NR, Wetterbom A, Zuccolo A, Lin YC, Scofield DG, et al. The Norway spruce genome sequence and conifer genome evolution. Nature. 2013;497(7451):579–84. 23698360 10.1038/nature12211

[93] Oksanen J. Vegan : Community Ecology Package. 2010. http://vegan.r-Forge.r-Project.org/ , https://cir.nii.ac.jp/crid/1570291225091856896.

[94] Oksanen J, Simpson GL, Blanchet FG, Kindt R, Legendre P, Minchin PR, O’Hara RB, et al. Vegan: Community Ecology Package. 2025. https://CRAN.R-project.org/package=vegan.

[95] Oliveira PH, Touchon M, Cury J, Rocha EPC. The chromosomal organization of horizontal gene transfer in bacteria. Nat Commun. 2017;8(1):841. 29018197 10.1038/s41467-017-00808-w PMC5635113

[96] Ondov BD, Treangen TJ, Melsted P, Mallonee AB, Bergman NH, Koren S, et al. “Mash: Fast Genome and Metagenome Distance Estimation Using MinHash.” Genome Biol. 2016;17(1):1–14. 27323842 10.1186/s13059-016-0997-x PMC4915045

[97] Pan Y, Birdsey RA, Fang J, Houghton R, Kauppi PE, Kurz WA, et al. A large and persistent carbon sink in the world’s forests. Science (New York, NY). 2011;333(6045):988–93. 10.1126/science.1201609 21764754

[98] Passel MWJV, Marri PR, Ochman H. The emergence and fate of horizontally acquired genes in *Escherichia coli*. PLoS Comput Biol. 2008;4(4):e1000059. 18404206 10.1371/journal.pcbi.1000059 PMC2275313

[99] Pimentel D, Harvey C, Resosudarmo P, Sinclair K, Kurz D, McNair M, et al. “Environmental and Economic Costs of Soil Erosion and Conservation Benefits.” Science. 1995;267(5201):1117–23. 17789193 10.1126/science.267.5201.1117

[100] Potter SC, Luciani A, Eddy SR, Park Y, Lopez R, Finn RD. HMMER web server: 2018 update. Nucleic Acids Res. 2018;46(W1):W200-204. 29905871 10.1093/nar/gky448 PMC6030962

[101] Putkinen A, Siljanen HMP, Laihonen A, Paasisalo I, Porkka K, Tiirola M, et al. New insight to the role of microbes in the methane exchange in trees: evidence from metagenomic sequencing. New Phytol. 2021;231(2):524–36. 33780002 10.1111/nph.17365

[102] Ramos JL, Martínez-Bueno M, Molina-Henares AJ, Terán W, Watanabe K, Zhang X, et al. The TetR Family of Transcriptional Repressors. Microbiol Mol Biol Rev. 2005;69(2):326–56. 15944459 10.1128/MMBR.69.2.326-356.2005 PMC1197418

[103] R Core Team. R: A Language and Environment for Statistical Computing. Vienna, Austria: R Foundation for Statistical Computing. 2024. https://www.R-project.org/.

[104] Redford AJ, Bowers RM, Knight R, Linhart Y, Fierer N. The ecology of the phyllosphere: geographic and phylogenetic variability in the distribution of bacteria on tree leaves. Environ Microbiol. 2010;12(11):2885–93. 20545741 10.1111/j.1462-2920.2010.02258.x PMC3156554

[105] Riley R, Bowers RM, Camargo AP, Campbell A, Egan R, Eloe-Fadrosh EA, et al. Terabase-scale coassembly of a tropical soil microbiome. Microbiol Spectr. 2023;11(4):e00200–23. 10.1128/spectrum.00200-23. 10.1128/spectrum.00200-23 PMC10434106 37310219

[106] Roberson EB, Firestone MK. Relationship between desiccation and exopolysaccharide production in a soil *Pseudomonas* sp. Appl Environ Microbiol. 1992;58(4):1284–91. 16348695 10.1128/aem.58.4.1284-1291.1992 PMC195588

[107] Rodríguez-Rodríguez JC, Bergeron Y, Kembel SW, Fenton NJ. Dominance of coniferous and broadleaved trees drives bacterial associations with boreal feather mosses. Environ Microbiol. 2022;24(8):3517–28. 35416394 10.1111/1462-2920.16013

[108] Rodriguez-Valera F, Martin-Cuadrado A-B, Rodriguez-Brito B, Lejla Pašić T, Thingstad F, Rohwer F, et al. Explaining Microbial Population Genomics through Phage Predation. Nat Rev Microbiol. 2009;7(11):828–36. 19834481 10.1038/nrmicro2235

[109] Rousk K, Jones DL, DeLuca TH. Moss-Cyanobacteria Associations as Biogenic Sources of Nitrogen in Boreal Forest Ecosystems. Front Microbiol. 2013;4. 10.3389/fmicb.2013.00150. 10.3389/fmicb.2013.00150 PMC3683619 23785359

[110] Roux S, Camargo AP, Coutinho FH, Dabdoub SM, Dutilh BE, Nayfach S, Tritt A. iPHoP: An Integrated Machine-Learning Framework to Maximize Host Prediction for Metagenome-Assembled Virus Genomes. bioRxiv. 2022. 10.1101/2022.07.28.501908. 10.1371/journal.pbio.3002083 PMC10155999 37083735

[111] Roux S, Camargo AP, Coutinho FH, Dabdoub SM, Dutilh BE, Nayfach S, Tritt A. iPHoP: An Integrated Machine Learning Framework to Maximize Host Prediction for Metagenome-Derived Viruses of Archaea and Bacteria. PLoS Biol. 2023;21(4):e3002083. 10.1371/journal.pbio.3002083 PMC10155999 37083735

[112] Saak CC, Dinh CB, Dutton RJ. Experimental approaches to tracking mobile genetic elements in microbial communities. FEMS Microbiol Rev. 2020;44(5):606–30. 32672812 10.1093/femsre/fuaa025 PMC7476777

[113] Saary P, Mitchell AL, Finn RD. Estimating the quality of eukaryotic genomes recovered from metagenomic analysis with EukCC. Genome Biol. 2020;21(1):244. 32912302 10.1186/s13059-020-02155-4 PMC7488429

[114] Saraiva JP, Bartholomäus A, Toscan RB, Baldrian P, da Rocha UN. “Recovery of 197 Eukaryotic Bins Reveals Major Challenges for Eukaryote Genome Reconstruction from Terrestrial Metagenomes.” Mol Ecol Resour. 2023;23(5):1066–76. 36847735 10.1111/1755-0998.13776

[115] Seidl R, Thom D, Kautz M, Martin-Benito D, Peltoniemi M, Vacchiano G, et al. Forest disturbances under climate change. Nat Clim Change. 2017;7(6):395–402. 10.1038/nclimate3303 PMC5572641 28861124

[116] Seppey M, Manni M, Zdobnov EM. BUSCO: Assessing Genome Assembly and Annotation Completeness. In Methods in Molecular Biology, 2019;1962:227–45. Humana Press Inc. 10.1007/978-1-4939-9173-0_14 31020564

[117] Shaw J, Yu YW. Fast and robust metagenomic sequence comparison through sparse chaining with Skani. Nat Methods. 2023;20(11):1661–5. 37735570 10.1038/s41592-023-02018-3 PMC10630134

[118] Shkoporov AN, Hill C. Bacteriophages of the Human Gut: The ‘Known Unknown’ of the Microbiome. Cell Host Microbe. 2019;25(2):195–209. 30763534 10.1016/j.chom.2019.01.017

[119] Song W, Wemheuer B, Zhang S, Steensen K, Thomas T. MetaCHIP: Community-Level Horizontal Gene Transfer Identification through the Combination of Best-Match and Phylogenetic Approaches. Microbiome. 2019;7(1). 10.1186/s40168-019-0649-y. 10.1186/s40168-019-0649-y PMC6399960 30832740

[120] Takeda K. Characteristics of a nitrogen-fixing methanotroph, *Methylocystis* T-1. Antonie Van Leeuwenhoek. 1988;54(6):521–34. 3148292 10.1007/BF00588388

[121] Tatum LA. “The Southern Corn Leaf Blight Epidemic: A New Race of the Fungus Helminthosporium Maydis Threatens Domestic Prices and Corn Reserves for Export.” Science. 1971;171(3976):1113–6. 17777595 10.1126/science.171.3976.1113

[122] Vacher C, Hampe A, Porté AJ, Sauer U, Compant S, Morris CE. The Phyllosphere: Microbial Jungle at the Plant–climate Interface. Annu Rev Ecol Evol Syst. 2016;47(1):1–24.

[123] Van Dongen S. “Graph Clustering Via a Discrete Uncoupling Process.” SIAM J Matrix Anal Appl. 2008;30(1):121–41.

[124] Vorholt JA. “Microbial Life in the Phyllosphere.” Nat Rev Microbiol. 2012. 10.1038/nrmicro2910. 23154261 10.1038/nrmicro2910

[125] Wang W, Schalamun M, Morales-Suarez A, Kainer D, Schwessinger B, Lanfear R. Assembly of chloroplast genomes with long- and short-read data: a comparison of approaches using *Eucalyptus pauciflora* as a test case. BMC Genomics. 2018;19(1):977. 30594129 10.1186/s12864-018-5348-8 PMC6311037

[126] Warner DF, Ndwandwe DE, Abrahams GL, Kana BD, Machowski EE, Venclovas C, et al. Essential Roles for imuA’- and imuB-Encoded Accessory Factors in DnaE2-Dependent Mutagenesis in Mycobacterium Tuberculosis. Proc Natl Acad Sci USA. 2010;107(29):13093–8. 20615954 10.1073/pnas.1002614107 PMC2919956

[127] West PT, Probst AJ, Grigoriev IV, Thomas BC, Banfield JF. Genome-reconstruction for eukaryotes from complex natural microbial communities. Genome Res. 2018;28(4):569–80. 29496730 10.1101/gr.228429.117 PMC5880246

[128] Wickham H. ggplot2: Elegant Graphics for Data Analysis. New York: Springer-Verlag; 2016.

[129] Yamaguchi Y, Park J-H, Inouye M. Toxin-antitoxin systems in bacteria and archaea. Annu Rev Genet. 2011;45(1):61–79. 22060041 10.1146/annurev-genet-110410-132412

[130] Yu G, Smith DK, Zhu H, Guan Yi, Lam T-Y. Ggtree : an R package for visualization and annotation of phylogenetic trees with their covariates and other associated data. Methods Ecol Evol. 2017;8(1):28–36. Edited by Greg McInerny.

[131] Zheng J, Ge Q, Yan Y, Zhang X, Huang Le, Yin Y. “dbCAN3: Automated Carbohydrate-Active Enzyme and Substrate Annotation.” Nucleic Acids Res. 2023;51(W1):W115–21. 37125649 10.1093/nar/gkad328 PMC10320055

