# Supplementary material for: Host species and geographic location shape microbial diversity and functional potential in the conifer needle microbiome
Source: Microbiome. 2025 Oct 30;13:222. doi: 10.1186/s40168-025-02271-y (PMC12574031; doi:10.1186/s40168-025-02271-y)
Supplement: Supplementary file 2 — Supplementary Material 2: Table 1. MAG counts by genome type, host species and metagenomic assembly type. Co‑assemblies were generated with MetaHipMer 2 [105], whereas individual metagenomes were assembled with metaSPAdes [91]. Species-level OTUs were defined using a 95% average nucleotide identity (ANI) threshold, for both bacterial and eukaryotic MAGs. The eukaryotic total counts include MAGs derived from host and insect DNA (10 MAGs total). MAG quality is reported in three numerical categories: ≥ 90 % completeness with ≤ 5 % contamination, 50–90 % completeness with ≤ 10 % contamination, and < 50 % completeness, because no community‑endorsed standard yet exists for assigning eukaryotic MAGs to the high‑, medium‑, and low‑quality (HQ/MQ/LQ) categories defined by the bacterial MIMAG guidelines [13]. [file 40168_2025_2271_MOESM2_ESM.pdf]

Domain	Conifer species	Assembler	Total Genomes	Species OTUs	>= 90% Completeness <= 5% Contamination	>= 50% Completeness <= 10% Contamination	< 50% Completeness
Bacteria	Engelmann spruce	metaSPAdes	47	43	9	21	17
Bacteria	Limber pine	metaSPAdes	48	37	3	30	15
Bacteria	Douglas fir	metaSPAdes	26	24	4	8	14
Bacteria	Engelmann spruce	coassembly	92	92	9	17	66
Bacteria	Limber pine	coassembly	62	62	5	16	41
Bacteria	Douglas fir	coassembly	52	52	10	10	32
Eukaryota	Engelmann spruce	metaSPAdes	7	6	0	5	2
Eukaryota	Limber pine	metaSPAdes	16	11	2	10	4
Eukaryota	Douglas fir	metaSPAdes	9	5	1	5	3
Eukaryota	Engelmann spruce	coassembly	15	14	0	9	6
Eukaryota	Limber pine	coassembly	39	39	1	13	25
Eukaryota	Douglas fir	coassembly	34	32	3	13	18
Total			447	417	47	157	243
