# Supplementary figures and images for: Host species and geographic location shape microbial diversity and functional potential in the conifer needle microbiome
Source: Microbiome. 2025 Oct 30;13:222. doi: 10.1186/s40168-025-02271-y (PMC12574031; doi:10.1186/s40168-025-02271-y)

A

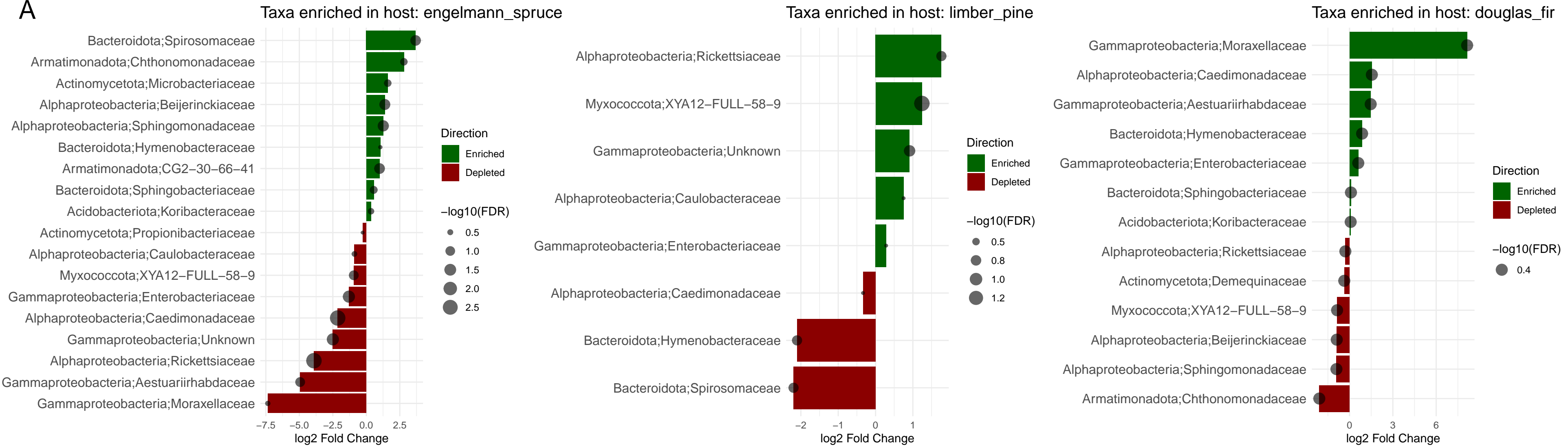

B

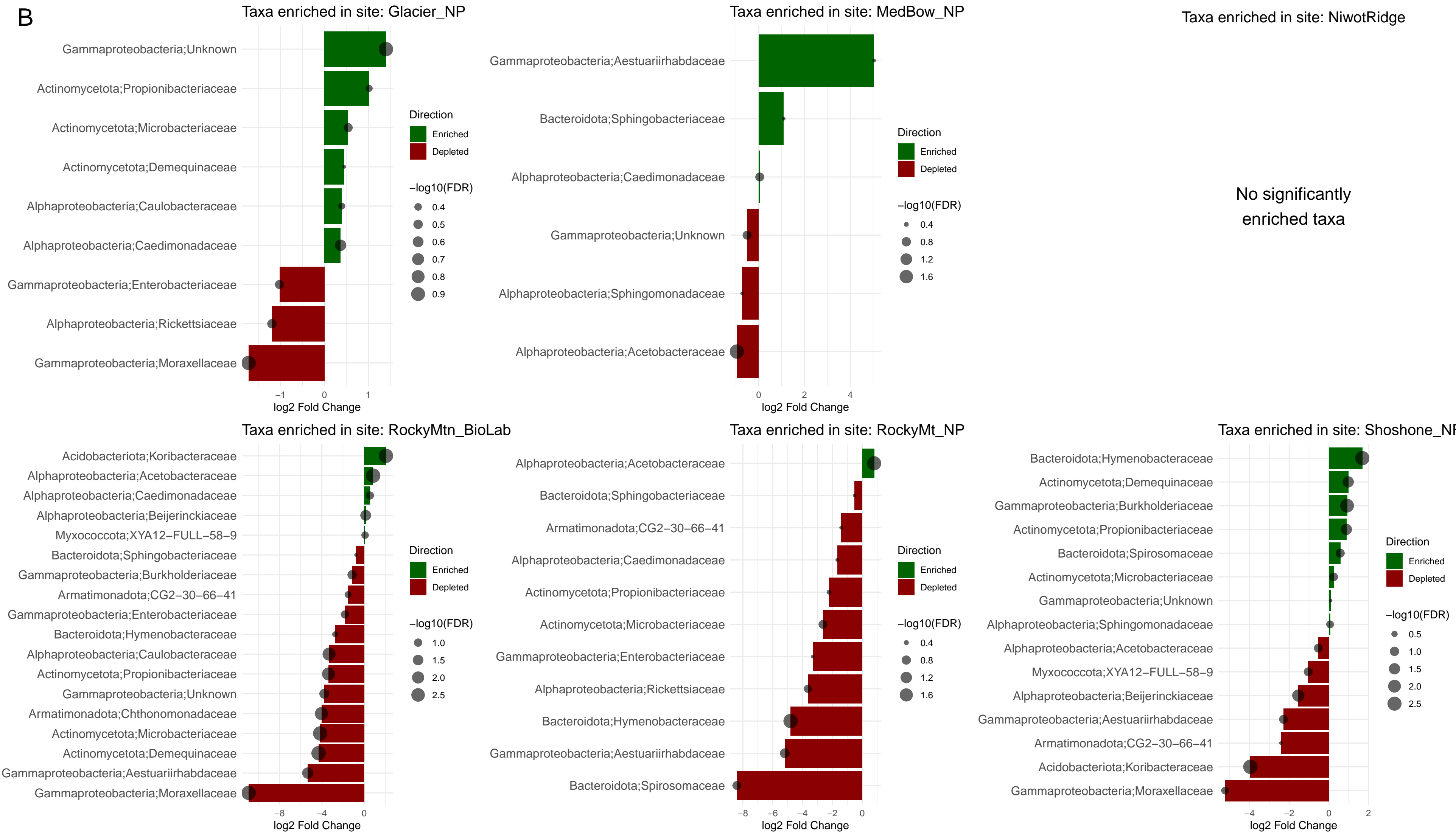

Supplement: Supplementary file 4 — Supplementary Material 4: Figure 2: Differential abundance of bacterial taxa across conifer host plants (A) and sampling sites (B) based on DESeq2 analysis. Each panel shows taxa significantly (FDR < 0.01) enriched (green bars) or depleted (red bars) in the target host/site compared to all other samples. Bars represent log2 fold change, with positive values indicating enrichment and negative values indicating depletion in the target condition. The size of black dots corresponds to statistical significance (-log10 of FDR), with larger dots indicating higher significance. Only the top differentially abundant taxa based on fold change magnitude are displayed in each panel. [file 40168_2025_2271_MOESM4_ESM.pdf]

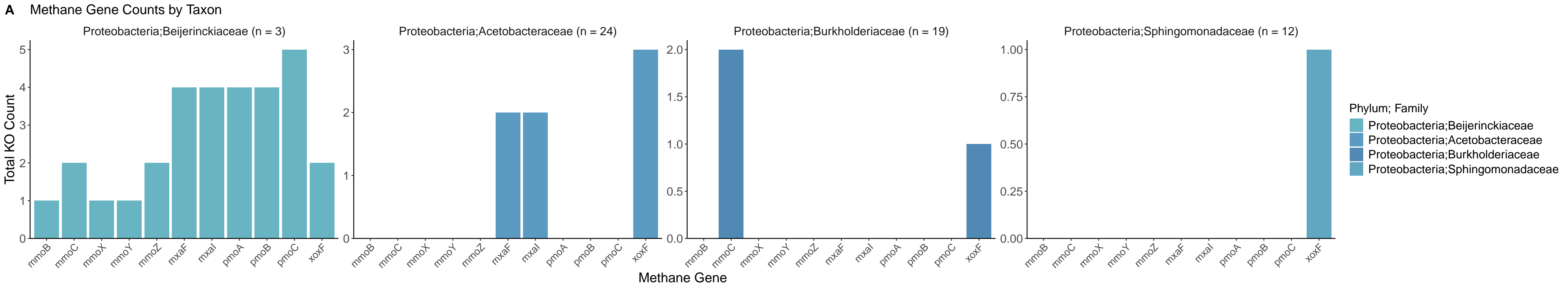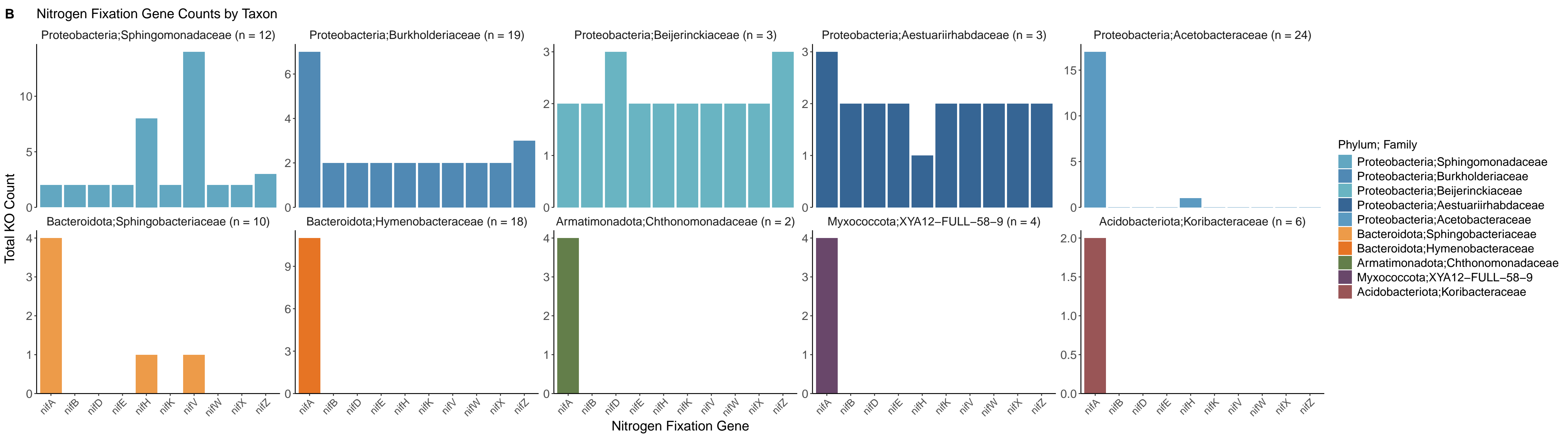

Supplement: Supplementary file 5 — Supplementary Material 5: Figure 3. Distribution of methane oxidation and nitrogen fixation genes across bacterial taxa. (A) Methane oxidation gene counts by taxon. Each bar represents the total number of KOs (KEGG Orthologs) for each methane oxidation gene (pmoA, pmoB, pmoC, mmoX, mmoY, mmoZ, mmoB, mmoC, mxaF) within each taxonomic group. Taxa are ordered by gene coverage (proportion of genes present) and total KO count. The number of genomes (n) for each taxon is indicated in parentheses. Colors represent different taxonomic groups. (B) Nitrogen fixation gene counts by taxon. Each bar represents the total number of KOs for each nitrogen fixation gene (nifH, nifD, nifK, nifE, nifN, nifB, nifQ, nifF, nifJ, nifA, nifL, anfG, anfD, anfK, nifM, nifS, nifU, nifV, nifW, nifZ, nifX, nifY) within each taxonomic group. Taxa are ordered by gene coverage and total KO count. The number of genomes (n) for each taxon is indicated in parentheses. [file 40168_2025_2271_MOESM5_ESM.pdf]

A

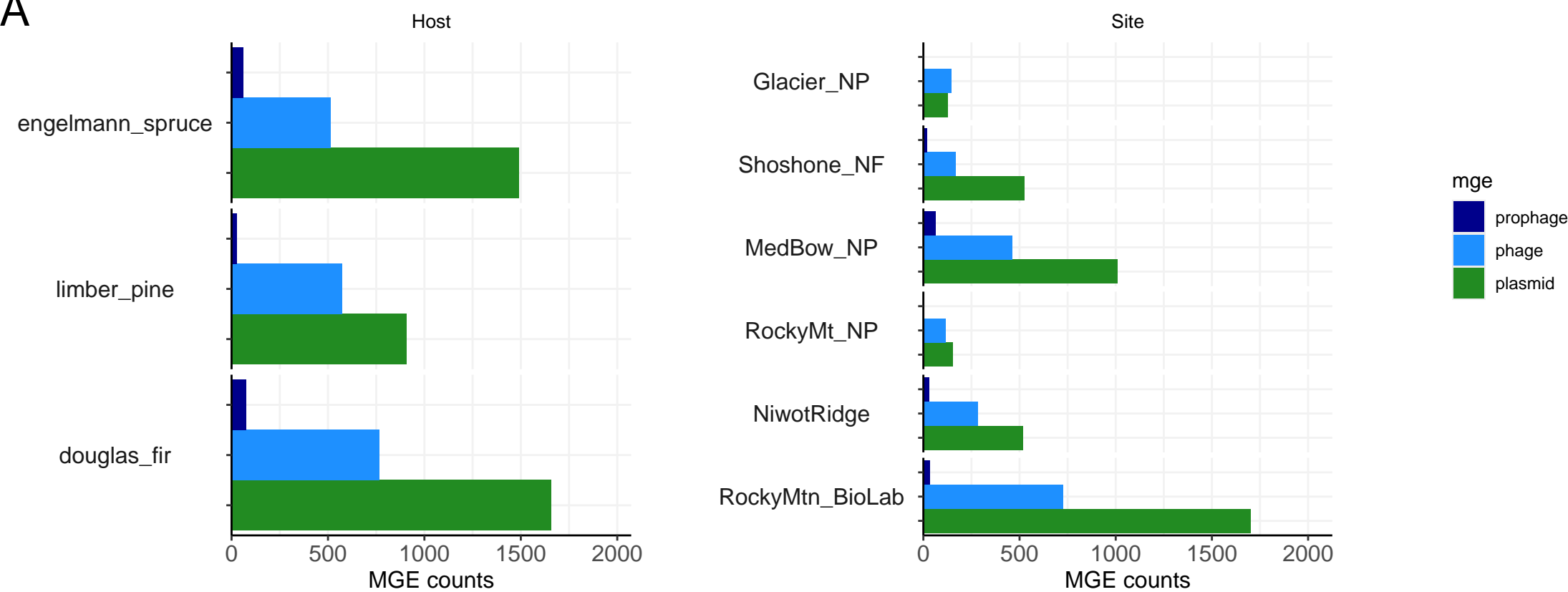

B

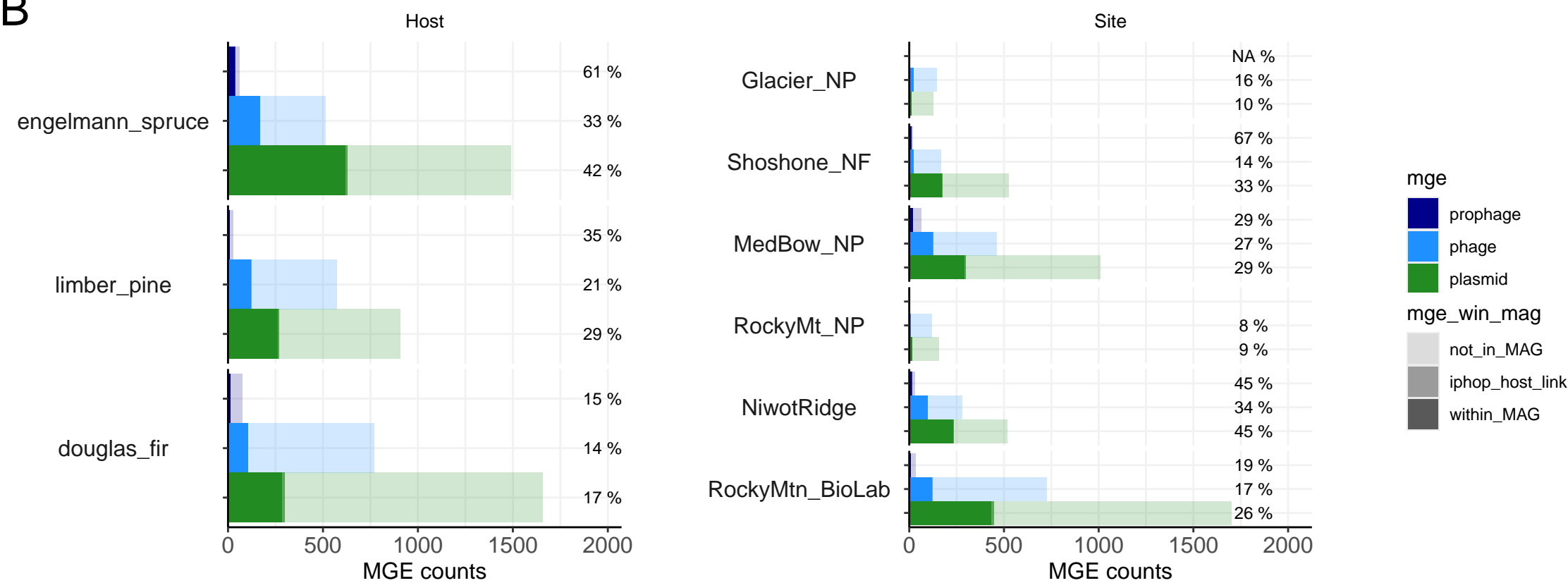

Supplement: Supplementary file 7 — Supplementary Material 7: Figure 5. Counts of MGEs greater than 5kb, normalized by total assembly size and total number of metagenomes per group. (A) Totals grouped by host and MGE type. (B) Totals grouped by site and MGE type, where shading represents those MGEs observed in a MAG and assigned to a host, those MGEs pulled into a MAG via iPhoP [111], and those MGEs that could not be assigned to a host MAG. [file 40168_2025_2271_MOESM7_ESM.pdf]

A

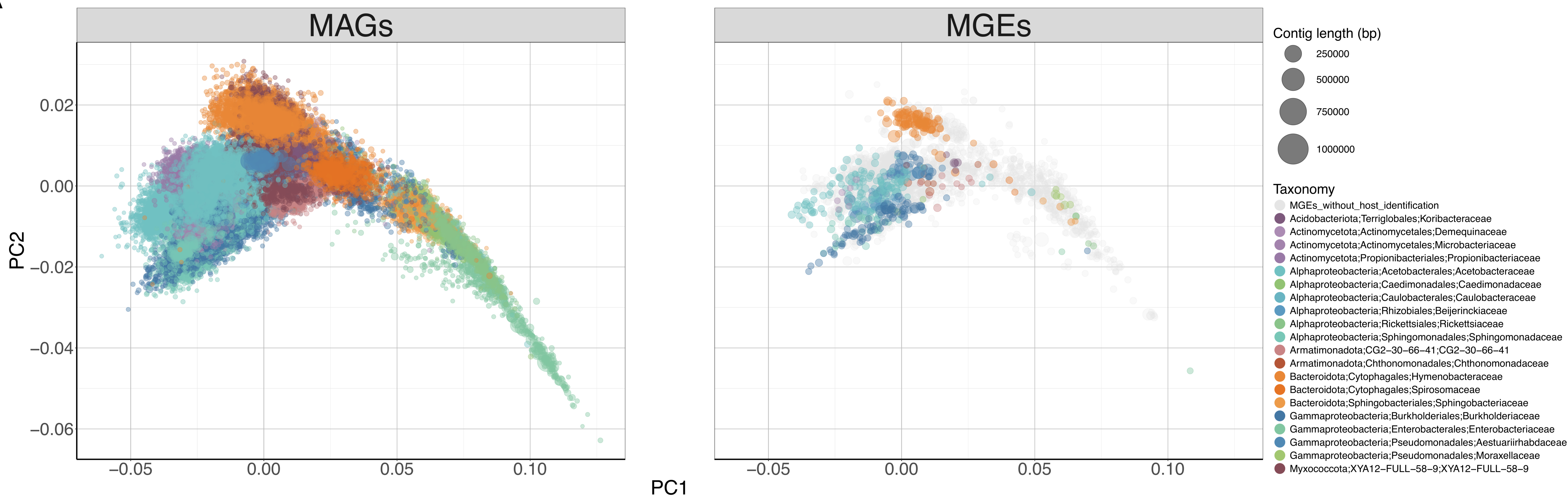

B

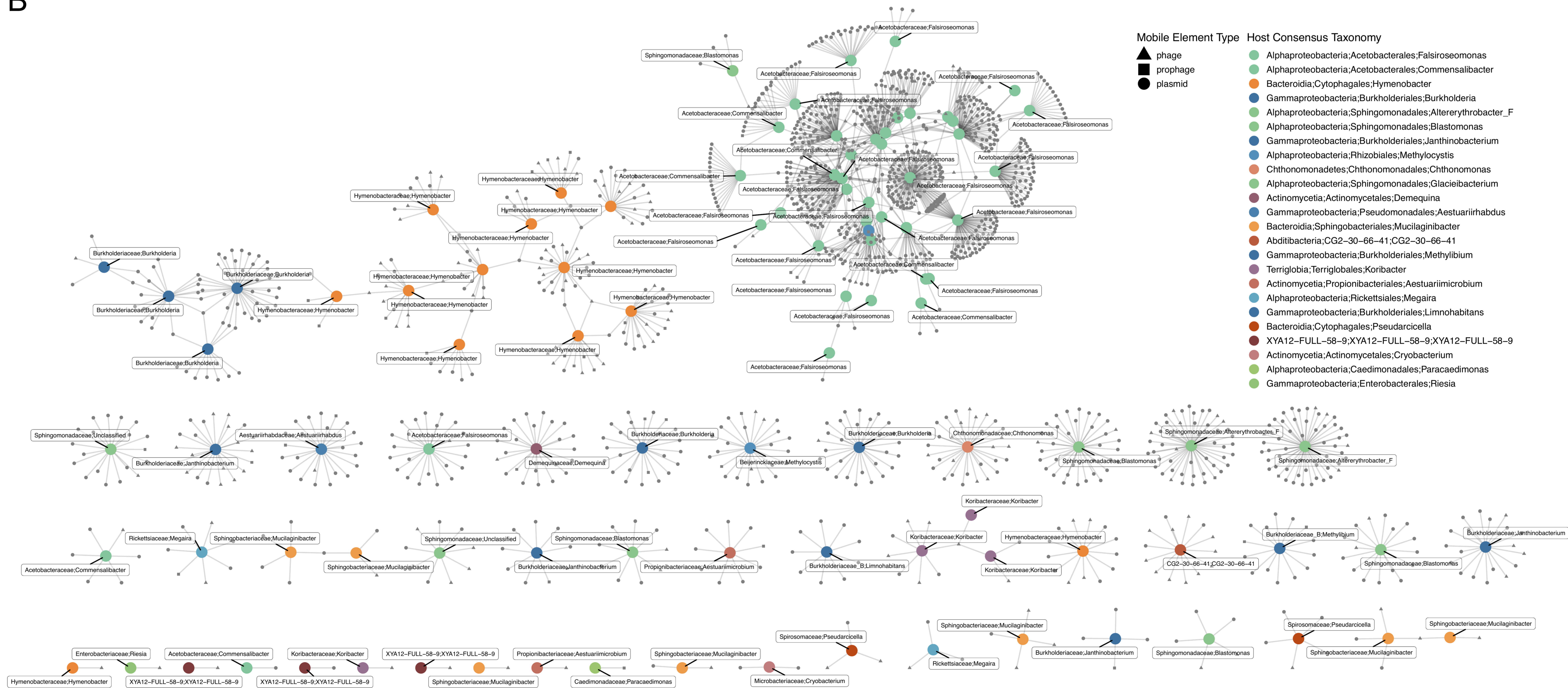

Supplement: Supplementary file 8 — Supplementary Material 8: Figure 6. Mobile genetic element analysis and virus-host network in the phyllosphere microbiome. (A) Principal component analysis (PCA) of tetranucleotide frequencies (TNF) of mobile genetic elements (MGEs) identified from metagenome-assembled genomes (MAGs). The plot illustrates the clustering of MGEs based on nucleotide composition patterns, with axes representing the first two principal components (PC1 and PC2). Each point represents an individual MGE, with colors indicating family level taxonomic identities. (B) Virus-host network visualization depicting predicted relationships between viral populations and their bacterial/archaeal hosts in the phyllosphere. Large colored nodes represent MAG hosts and smaller nodes represent their MGEs including phages, prophages and plasmids. [file 40168_2025_2271_MOESM8_ESM.pdf]

# Number of Cross-Taxon HGTs by Taxonomic Rank

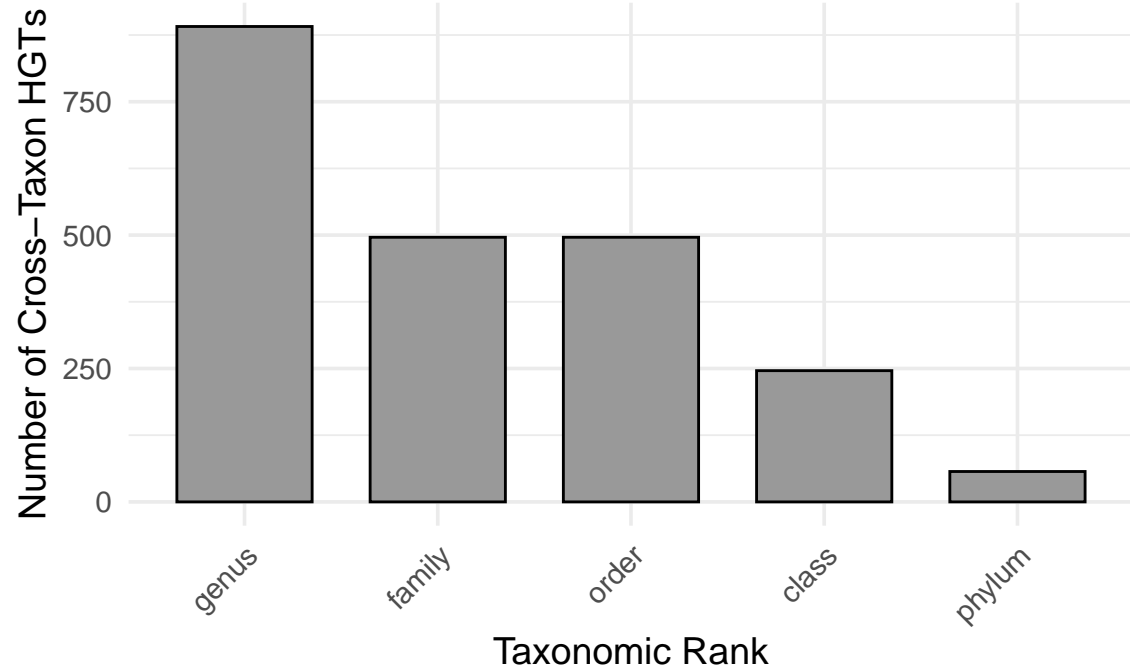

Supplement: Supplementary file 9 — Supplementary Material 9: Figure 7. Number of cross-taxon Horizontal Gene Transfers (HGTs) across taxonomic ranks. Bar plot showing the total number of inferred cross-taxon horizontal gene transfers (HGTs) identified at five different taxonomic ranks: genus, family, order, class, and phylum. The majority of HGTs occur between closely related taxa (e.g., genus and family), with decreasing numbers observed at higher taxonomic levels. [file 40168_2025_2271_MOESM9_ESM.pdf]
