# Supplementary material for: Host species and geographic location shape microbial diversity and functional potential in the conifer needle microbiome
Source: Microbiome. 2025 Oct 30;13:222. doi: 10.1186/s40168-025-02271-y (PMC12574031; doi:10.1186/s40168-025-02271-y)
Supplement: Supplementary file 6 — Supplementary Material 6: Figure 4. Distribution of carbohydrate substrate degradation potential across bacterial taxa. The stacked bar plot shows the substrate degradation potential across different bacterial taxonomic groups. Each bar represents a taxonomic group (phylum;family), with Y-axis labels colored by taxonomy. The X-axis shows the normalized count of substrate-degrading enzymes per genome, indicating the average number of enzymes each genome in that taxonomic group possesses for each substrate type. Colors represent different substrate types, ordered from plant cell wall components (blues) through host glycans (greens) to various polysaccharides (reds, oranges, and purples). Taxa are ordered by their total normalized enzyme count, with those at the bottom having the fewest substrate-degrading enzymes and those at the top having the most diverse substrate utilization potential. This visualization reveals substrate specialization patterns across bacterial lineages, highlighting which taxonomic groups are specialized for specific substrate types versus those with broader degradation capabilities. [file 40168_2025_2271_MOESM6_ESM.pdf]

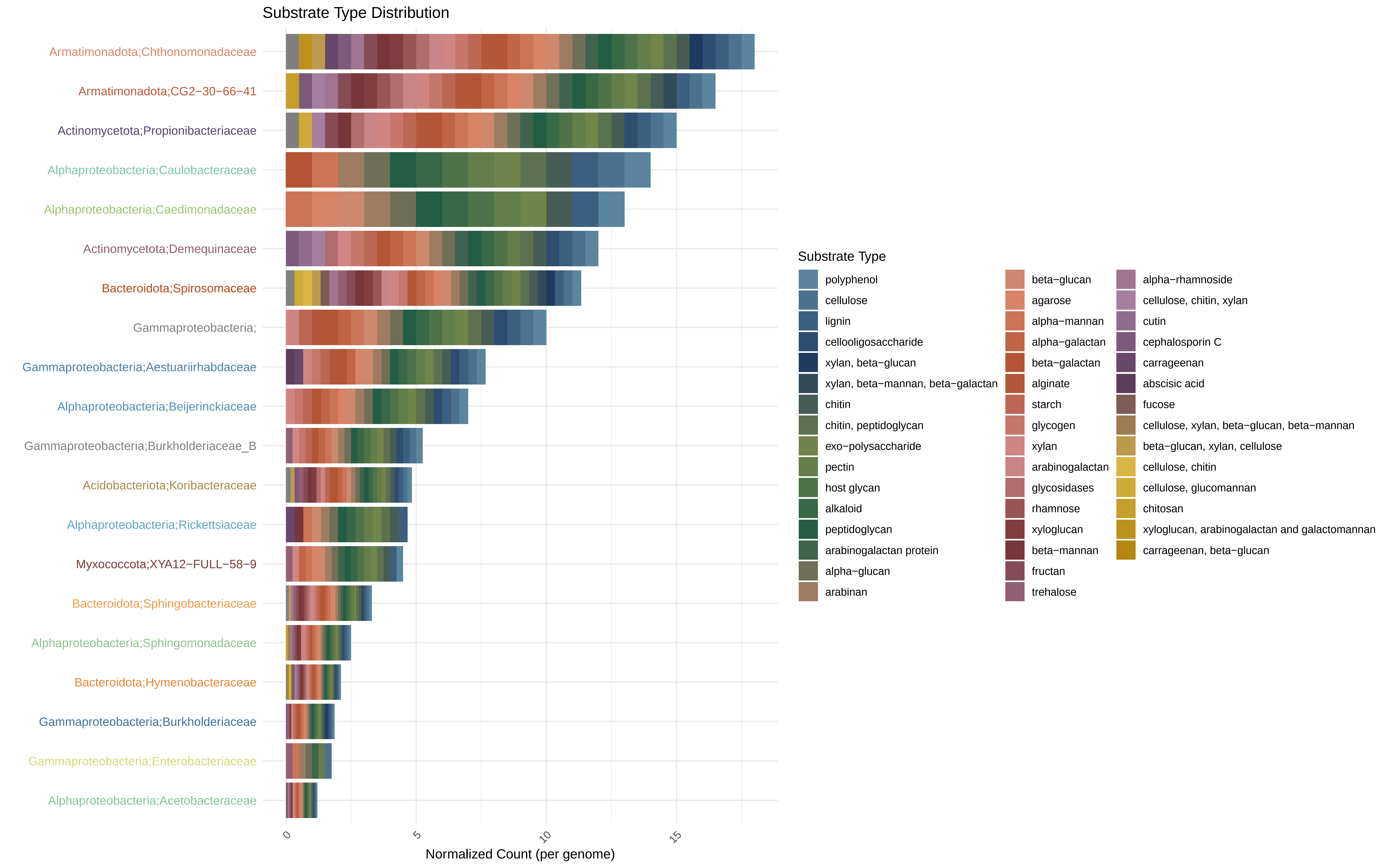

Substrate Type Distribution
Armatimonadota;Chthonomonadaceae
Armatimonadota;CG2−30−66−41
Actinomycetota;Propionibacteriaceae
Alphaproteobacteria;Caulobacteraceae
Alphaproteobacteria;Caedimonadaceae
Actinomycetota;Demequinaceae
Bacteroidota;Spirosomaceae
Gammaproteobacteria;
Gammaproteobacteria;Aestuariirhabdaceae
Alphaproteobacteria;Beijerinckiaceae
Gammaproteobacteria;Burkholderiaceae_B
Acidobacteriota;Koribacteraceae
Alphaproteobacteria;Rickettsiaceae
Myxococcota;XYA12−FULL−58−9
Bacteroidota;Sphingobacteriaceae
Alphaproteobacteria;Sphingomonadaceae
Bacteroidota;Hymenobacteraceae
Gammaproteobacteria;Burkholderiaceae
Gammaproteobacteria;Enterobacteriaceae
Alphaproteobacteria;Acetobacteraceae
0
5
10
15
Normalized Count (per genome)
Substrate Type
polyphenol
cellulose
lignin
cellooligosaccharide
xylan, beta−glucan
xylan, beta−mannan, beta−galactan
chitin
chitin, peptidoglycan
exo−polysaccharide
pectin
host glycan
alkaloid
peptidoglycan
arabinogalactan protein
alpha−glucan
arabinan
beta−glucan
agarose
alpha−mannan
alpha−galactan
beta−galactan
alginate
starch
glycogen
xylan
arabinogalactan
glycosidases
rhamnose
xyloglucan
beta−mannan
fructan
trehalose
alpha−rhamnoside
cellulose, chitin, xylan
cutin
cephalosporin C
carrageenan
abscisic acid
fucose
cellulose, xylan, beta−glucan, beta−mannan
beta−glucan, xylan, cellulose
cellulose, chitin
cellulose, glucomannan
chitosan
xyloglucan, arabinogalactan and galactomannan
carrageenan, beta−glucan
